# Developing policy on sugar-sweetened beverages for children and adolescents in China: a qualitative study of stakeholder views and perceptions

**DOI:** 10.1136/bmjopen-2025-098746

**Published:** 2025-12-12

**Authors:** Yue Suo, Jiajie Zang, Jia Wang, Qianqian Shen, Qian Long

**Affiliations:** 1 Global Health Research Centre, Duke Kunshan University, Kunshan, Jiangsu, China; 2 School of Public Health, Fudan University, Shanghai, China; 3 Shanghai Municipal Center for Disease Control and Prevention, Shanghai, China; 4 Yuzhong District Center for Disease Control and Prevention, Chongqing, China

**Keywords:** Child, Health policy, NUTRITION & DIETETICS, Obesity, China

## Abstract

**Abstract:**

**Objective:**

To explore stakeholder perceptions on sugar-sweetened beverage (SSB) policies for Chinese children and adolescents and facilitators and challenges for policy implementation.

**Design:**

This study followed the sector governance analysis framework, which included three steps: context analysis, mapping stakeholders and stakeholder analysis. Context analysis comprised policy and literature reviews of existing domestic and international measures, complemented by expert consultation to clarify the policy context and identify relevant stakeholders. Guided by these insights, we mapped stakeholders for key informant interviews, in-depth interviews and focus group discussions to explore stakeholders’ perceptions of SSB policies. Qualitative data were collected and analysed through a thematic analysis approach.

**Setting:**

Shanghai and Chongqing, China, July to August 2022.

**Participants:**

37 stakeholders including policymakers, nutrition experts, industry and consumers (primary caregivers of children and adolescents aged 6–17 years).

**Results:**

Context and stakeholder analyses indicated rising SSB consumption among Chinese youth since 2000. Qualitative interviews reflected the absence of national policies due to inadequate policymaker awareness. Although policymakers and nutrition experts supported SSB policies, consumers were worried about their personal choices being affected and the industry feared innovation challenges and profit loss. Multiple stakeholders mentioned that a comprehensive national standard is lacking, which is needed to facilitate national policy roll-out. An initial focus on health education is suggested to raise awareness among policymakers and consumers to foster a supportive environment for SSB policy development.

**Conclusions:**

Although SSB intake is rising among Chinese children, policymakers’ insufficient awareness and the lack of national standards hinder SSB policy development and implementation. Strategies that raise health knowledge and awareness among policymakers and consumers should be prioritised for now to assist future introduction of SSB standards and related policies.

**STRENGTHS AND LIMITATIONS OF THIS STUDY**

- This study provides an empirical, multi-stakeholder analysis of sugar-sweetened beverage (SSB) policies for children and adolescents in the Chinese context, addressing the evidence gap on perspectives of policymakers, industry stakeholders, nutrition experts and consumers (primary caregivers of children and adolescents).
- The study adapted the sector governance analysis framework to integrate context analysis, stakeholder mapping and stakeholder analysis (including key informant interviews, in-depth interviews and focus group discussions), which ensures the coherence between research questions, evidence sources and policy interpretation.
- The inclusion of policymakers, nutrition experts, industry stakeholders and consumers enabled a comprehensive assessment of political, technical, commercial and behavioural factors influencing potential SSB policy introduction for children and adolescents in China.
- All consumer participants (primary caregivers of children and adolescents) were recruited from urban communities in two municipalities, and rural populations were not included, which may limit the generalisability of consumer-level findings at the national level.
- Because of travel restrictions during the COVID-19 pandemic, data collection procedures at the two study sites differed and some data were collected through remote communication portals, which may have influenced the results.

## Introduction

 According to the WHO, sugar-sweetened beverages (SSBs) are defined as beverages containing free sugars,^1^ and they are calorie-dense foods with limited nutritional merit. SSBs are a major source of free sugars in the diet, and SSBs are increasingly consumed worldwide,^2 3^ especially among children and adolescents. Many studies have shown that excess consumption of SSBs could increase the risk of childhood obesity,^4^,^7^ dental caries^8^ and type 2 diabetes mellitus (T2DM),^9^ which could trigger other non-communicable diseases (NCDs) and increase the risk of mortality.^9 10^ Some international communities have published guidelines suggesting policies including taxation, labelling or marketing restrictions as effective interventions to reduce SSB consumption for NCD prevention, especially in low-income and middle-income countries (LMICs).^11 12^ The effect of such policies has shown a significant reduction in purchases.^13 14^ For instance, in Mexico, 2 years after introducing an SSB tax, the purchase of taxed drinks decreased by 7.6%.^15^

China has witnessed a growing prevalence of T2DM and childhood obesity over the past decades, with an alarming increase in free-sugar consumption through SSBs, and children and adolescents are the greatest consumers.^16^ On average, school-age youth (aged 6–17 years) consume SSBs 3.9 times per week,^16^ whereas the 2016 national cross-sectional survey in urban China showed that 27% of children and 48% of adolescents consumed more than one serving (250 mL) of SSBs every day.^17^ Nevertheless, China has advised the harm of excess SSB intake through advocacy guidelines, school sale bans and shelf warning labels. China’s national dietary guide recommended the importance of drinking water, as ‘SSBs are not alternatives to water’.^18^ Furthermore, to implement the ‘Health Diet Initiative’ of ‘Healthy China Initiative (2019–2030)’,^19^ multiple national departments (including the National Health Commission, Ministry of Education, State Administration of Market Regulation and the General Administration of Sport) enacted the ‘Guidelines for Building Healthy and Nutrition-Aware Schools’ in 2021,^20^ stating that “No convenience stores, supermarkets, or other food retailing facilities shall be set up in schools, and no alcoholic beverages or food high in salt, sugar, fat shall be sold… Advertisements of SSBs are not allowed”. Meanwhile, Shenzhen was the first city in China to release a standard for setting health warning signs for carbonated SSBs in 2020, specifying that shelves displaying SSBs should be equipped with standard health warning signs.

However, China has not announced a national policy directly discouraging SSB consumption in line with international recommendations, for instance, through taxation, labelling or marketing restrictions to encourage less purchase. Establishing a new policy framework requires consideration of factors such as geographical disparities, sociodemographic structure and economic development, as well as involvement of various stakeholders. Stakeholders with different decision-making power also hold diverse opinions, which may either facilitate or hinder policy introduction and implementation. Therefore, understanding stakeholder perspectives enables deeper insight into the policy environment, which is necessary for effective policy formulation and implementation. Considering China’s vast regional disparities in geography, demographic structure and economic development, which shape heterogeneous responses towards SSB policies,^21^ the feasibility of SSB policies in China remains unclear. According to a preliminary search, there has been a knowledge gap in studying stakeholders’ perspectives of SSB policy implementation in China. Therefore, this study aimed to explore policymakers, nutrition experts, industry representatives and consumers’ knowledge, attitudes and perceptions regarding SSB policies targeting children and adolescents in China, as well as the facilitators and challenges to implementing such policies.

## Methods

This study referenced the consolidated criteria for reporting qualitative research for organising findings (see online supplemental appendix 1E for the detailed checklist items).

### Conceptual framework

This qualitative study was underpinned by the sector governance analysis framework,^22^ which is suitable for the study context where research evidence on SSB policies for children and adolescents in China remains limited; thus, it provided a structured and systematic lens to explore the policy implementation environment. The framework directly informed the study design by guiding data collection and analysis through the following three steps: context analysis, mapping stakeholders and stakeholder analysis.

### Study setting

The study was conducted in two Chinese municipalities, Shanghai and Chongqing. The sites were chosen considering geographical and socio-economic disparities. Shanghai, a highly developed municipality in eastern China, had a gross domestic product (GDP) per capita of 27 372 USD in 2022.^23^ The prevalence of child overweight and obesity is 45.0% and 19.0% respectively.^24^ In contrast, Chongqing is a comparatively less developed municipality in western central China, with a GDP per capita of US$12 521 in 2022^23^ and child overweight and obesity prevalence of 9.6% and 17.0%, respectively^25^.

### Context analysis

Context analysis consisted of policy review and literature review. First, in the policy review, we defined SSB-related policies as those including measures that discourage intake of packaged SSBs. For example, through taxation, labelling (applying extra labels on SSB packages or retailers), restricting SSB supply (limiting SSB availability at specific locations), marketing restrictions (limiting media advertisements of SSBs) or health education campaigns (providing health knowledge to the public), this study focused on these five forms of SSB policies. International and Chinese policies were collected from the official websites of international organisations (WHO Global Health Observatory, United Nations Food and Agriculture Organisation and Global SSB Tax Database of the World Bank), related associations (World Cancer Research Fund NOURISH policy database, Global Food Research Programme by UNC-Chapel Hill and Bloomberg Philanthropies Food Policy Programme) and government agencies (National Health Commissions of China, State Administration for Market Regulation of China and Chinese Center for Disease Control and Prevention (CDC)).

To gain an understanding of the effectiveness of evidence-based interventions on the restriction of SSBs and their implementation challenges, we also conducted a rapid scoping literature review on SSB control strategies from other countries, with a focus on LMICs, as well as SSB consumption trends and sugar intake control strategies in China between January and April 2022. Search terms included ‘sugar-sweetened beverages’, ‘nutrition’, ‘child nutrition’, ‘beverage policy’, ‘taxation’ and ‘labelling’. Original research and programme reports written in English and Chinese and published from 2005 to 2022 from MEDLINE and Google Scholar were included. Additionally, a manual search was conducted to identify relevant grey reports and regional policies from Shenzhen and Shanghai according to expert consultation. The collected literature and policies were classified based on the policy content and target. We consulted a public health nutrition expert in child nutrition and nutrition policy planning (who was later formally interviewed as part of this study) about the search strategy and scope to ensure comprehensive coverage of relevant policy and literature sources.

Policy metrics were used to assess the policy environment in China. A data extraction form was developed to summarise key features of policies, including the title, issuing department, year of issue and policy content. These metrics were integrated to compare current SSB-related policies targeting children and adolescents in China.

Based on the context analysis results, we consulted the previous public health nutrition expert on potential stakeholders for qualitative interview. According to their suggestions, we identified potential stakeholders to approach for qualitative interviews and mapped them through listing out potential roles across four key sectors (policymakers, nutrition experts, industry and consumers).

### Qualitative interviews

#### Data collection

From July to September 2022, stakeholders from four sectors were interviewed, including policymakers, nutrition experts, industry and consumers (primary caregivers of children and adolescents aged 6–17). Policymakers and nutrition experts were engaged through key informant interviews, industry stakeholders through individual in-depth interviews, and consumers through focus group discussions (FGDs). All interviews and FGDs were conducted using semi-structured interview guides, which were developed based on the reviewed policies and literature, with a version each for policymakers and nutrition experts, industry stakeholders and consumers (see detailed interview guides in online supplemental appendix 1B).

The eligibility criteria for policymakers and nutrition experts were professionals in the field of child nutrition, food policy and marketing regulations. Policymakers and nutrition experts were approached purposefully through professional connections via phone call and WeChat (a commonly used social media platform in China) and participated in key informant interviews on knowledge and perceptions of SSB policies, facilitators and barriers of SSB policy implementation and SSB policy design in China. Additionally, policymakers and nutrition experts were asked about their opinion on the adaptability (considering both feasibility and effectiveness) of five specific types of SSB policies, including taxation, labelling, restricting SSB supply, restricting SSB marketing and advertising and health education. See detailed definitions of these policies in online supplemental appendix 1D.

Industry stakeholders specialised in research and development (R&D) and marketing from companies selling SSBs, artificially sweetened beverages (ASBs) or both SSB and ASBs were eligible to attend in-depth interviews, and they were recruited purposefully through professional connections via WeChat. Their interviews focused on knowledge and perceptions of SSB policies, China’s SSB market development and potential responses to SSB policies if implemented in China.

Consumers who participated in this study were primary caregivers of children aged 6–17, recruited through convenience sampling by community health centre workers. Residents living in the corresponding community were approached during their community health centre visits and through online chat groups and invited to participate. In each city, the recruited consumers were divided into two groups based on the age group of their children, one with primary caregivers of children aged 6–11 and the other group with primary caregivers of adolescents aged 12–17. In total, four FGDs were conducted (two in Shanghai and two in Chongqing). FGDs covered themes on knowledge and perceptions of SSB policies and SSB consumption habits.

One author (YS) with prior training in qualitative research at Duke Kunshan University conducted the interviews for this study. Before the data collection, YS conducted three mock interviews as preparation. Participants in Chongqing were interviewed through an online meeting portal (Tencent Meeting version 3.9.0) due to pandemic travel restrictions. FGDs in Chongqing were coordinated by local CDC staff. Besides, participants in Shanghai were interviewed in person either in their office (policymakers, nutrition experts and industry) or the community centre (consumers). Before the interview, participants received an information sheet that outlined the study aim, duration of the interview, confidentiality and ethical assurances. Specifically, no description or background information about SSB policies was provided to participants before the interviews to capture their awareness, knowledge and perceptions of SSB policies. If a participant showed unfamiliarity with SSB policies during the interview, YS then briefly described the policy concept to facilitate the discussion. At the beginning of the interview, participants provided their informed consent verbally to the interviewer (see detailed consent form scripts in online supplemental appendix 1C). All interviews were conducted in Mandarin Chinese, each interview lasted for 45−60 min and each focus group discussion lasted for 60 min. Most interviews were video recorded, except one with a policymaker who did not permit recording (detailed notes were taken during this interview).

#### Data analysis

All interviews were transcribed verbatim in Chinese using Lark Minutes (an automatic audio-transcript converter) and manually reviewed to ensure accuracy. Data were analysed using a thematic content approach.^26^ First, one analyst (YS) undertook familiarisation with the transcripts by reading transcripts repeatedly. Key points for the coding scheme were then identified after discussing with the author who was most experienced in qualitative research (QL). Second, structural codes were generated based on the interview guides, and emergent themes were developed. Third, the coding scheme was improved through memo writing on uncoded data, preliminary coded data and re-coded data. Fourth, all transcripts were coded in Chinese through NVivo version 12.6.1. Finally, translation from Chinese to English was completed by YS, with translation accuracy cross-checked through discussion with QL. Illustrative quotations were identified and presented in the Results section.

Stakeholder analysis was conducted to identify and understand key actors who are involved in a policy issue, domains including their interests, influence and relationships within the policy system.^27 28^ This enables the assessment of policy context by identifying stakeholder positions and their power dynamics, which is significant to evaluate the policy feasibility. In addition, a stakeholder analysis was conducted to assess each stakeholder interviewed (except for consumers, they were analysed as a whole group) through three dimensions: interest (stakeholder’s interest in introducing SSB policies for children and adolescents in China), power (stakeholder’s decision-making power) and position (whether a stakeholder supports or opposes SSB policies for Chinese children and adolescents).^29^ These dimensions were derived through triangulation of data sources, including interview transcripts, context analysis findings and discussion among the study team.

Quality and trustworthiness of the collected qualitative data interpretation were assured through triangulating findings in various ways, including cross-checking of results, review of relevant literature and policies, discussion of results and analysis among the study team and with the study site collaborators.

### Ethics approval

Ethical clearance was granted by the Institutional Review Board at Duke Kunshan University (Effective date 20 May 2022. Reference No. 2022YS039).

### Patient and public involvement

Consumers (caregivers of adolescents and children who consume SSBs) were not involved in the study design, but they engaged in disseminating the interviewee recruitment information, which accelerated the recruitment process during the study.

## Results

The results are presented in three parts. First, the context analysis outlines SSB consumption trends among children and adolescents in China, the existing national policy environment and relevant international experiences. Second, stakeholder mapping is presented to identify actors relevant to SSB policy implementation. Third, the stakeholder analysis integrates findings from the previous steps and findings from qualitative interviews and FGDs, including stakeholders’ knowledge, perceptions and perceived barriers and facilitators to policy adoption.

### Context analysis

#### Sugar-sweetened beverage consumption of children and adolescents in China

As part of the context analysis, national survey data and published literature were reviewed to describe consumption patterns. Literature shows that there has been a rising trend of SSB consumption in adolescents and children since 2000.^30^ First, China’s SSB production and sales have continued to grow, with beverage production exceeding 177.635 million tons in 2019.^31^ Literature proved that children and adolescents (aged 6–17) consumed SSBs the most. National survey data indicated that 66.6% of children and adolescents consumed SSBs at least once a week, and 9.6% consumed more than 250 mL per day on average. For Chinese adolescents (aged 13–17), the 95th percentile of free-sugar energy contribution ratio from SSBs is around 9.31%,^32^ which is close to the 10% upper limit of free-sugar energy contribution ratio suggested by the WHO.^33^

#### Sugar-sweetened beverage policy context for children and adolescents in China

The context analysis also reviewed existing policy measures in China (see table 1 for the current SSB-related policies in China). Although a national policy specifically targeting SSB intake is absent, there have been significant efforts to regulate SSB availability on school campuses. For instance, Shanghai initiated a ban on SSB sales in schools as early as 2008,^34^ and similar actions were adopted in Beijing in 2013.^35^ Such efforts have been extended with the ‘Oral Health Action Programme (2019–2025)’, advocating for reduced sugar consumption among students.^36^ The broader initiative, ‘Healthy China Initiative (2019–2030)’, aims to improve dietary habits,^19^ and it includes the ‘National Nutrition Plan (2017–2030)’ and enforces strict guidelines against the sale of high-sugar content items in schools.^37^ Moreover, Shenzhen has pioneered labelling SSBs, launching health warning labels that indicate the health risks of high sugar intake in 2020.^38^ This collective approach reflects China’s strategic efforts to combat diet-related health issues through policy interventions, educational reforms and regulatory measures targeting both schools and broader community settings.

**Table 1:** Current SSB-related policies for children and adolescents in China

Title	Issue department	Year	Content
Notice on standardising the provision of meals for students in primary and secondary schools and the establishment of supermarkets (grocery stores) in schools in Shanghai	Shanghai Municipal Education Commission	2008	Ban SSB sales in schools
Notice on further regulating the management of food and beverages in primary and secondary schools	Beijing Municipal Education Commission	2013	Ban SSB sales in schools
Healthy China 2030 Initiative	State Council of the PRC	2016	Healthy Diet Initiative
National Nutrition Plan (2017–2030)	State Council of the PRC	2017	The ‘San Jian (3 Reductions)’ including sugar reduction
Healthy Mouth Action Programme (2019–2025)	National Health Commission of the PRC	2019	Restrict supply of SSBs in school canteens
School Food Safety and Nutrition Health Management Regulations (2019)	Ministry of Education of the PRC	2019	Ban food retailing facilities in non-boarding schools, restrict SSB sales in schools
Guidelines for Building Healthy and Nutrition-Aware Schools	Health Commission, Ministry of Education, General Administration of Market Regulation, General Administration of Sports of the PRC	2021	Ban SSB advertising in schools
Dietary Guideline for Chinese Residents (2022)	Chinese Nutrition Society	2022	Recommend less or no intake of SSBs

PRC, People’s Republic of China; SSB, sugar-sweetened beverage.

#### SSB control measures in other countries

International approaches to controlling SSB consumption include SSB taxes, SSB labelling and restrictions on SSB supplies and marketing.^3 39^ Some studies have also discussed restricting SSB supplies at schools and retailers and promoting health education.^40 41^

SSB taxes are considered one of the ‘Tackling NCD Best Buys’ by the WHO, showing cost-effectiveness in LMICs.^11^ By 2023, over 50 countries and regions had implemented an SSB tax. Mexico witnessed a 9.7% drop in SSB purchases during the first 2 years of implementation,^15^ and the tax also induced a 4% increase in the sales of water and non-taxed beverages.^42^ The Chilean SSB tax also caused a 21.6% decrease in the monthly purchased volume of the higher-taxed SSBs, despite the change in overall SSB purchase volume being not significant.^43^

The WHO also recommended using front-of-package (FOP) labels and nutrition labels to encourage healthier diets. A Cochrane review identified moderate-certainty evidence that traffic-light labelling reduces SSB sales.^39^ After adopting the traffic light labelling in 2013, Ecuador witnessed a decline in high-sugar carbonated SSB purchases and an increase in low-sugar or sugar-free carbonated SSB purchases.^44^

Some countries have also begun restricting advertising and marketing to control SSB intake. For example, Chile introduced restrictions on television advertising campaigns on SSBs aimed at children under 14, resulting in 44% and 58% less unhealthy food and beverage advertising, respectively, on television after the law was implemented.^41^

Overall, the effect of these measures in LMICs varies, but they have generally shown a positive impact on reducing SSB consumption and increasing public health awareness.

### Stakeholder mapping of sugar-sweetened beverage policy implementation in China

**Figure 1:**
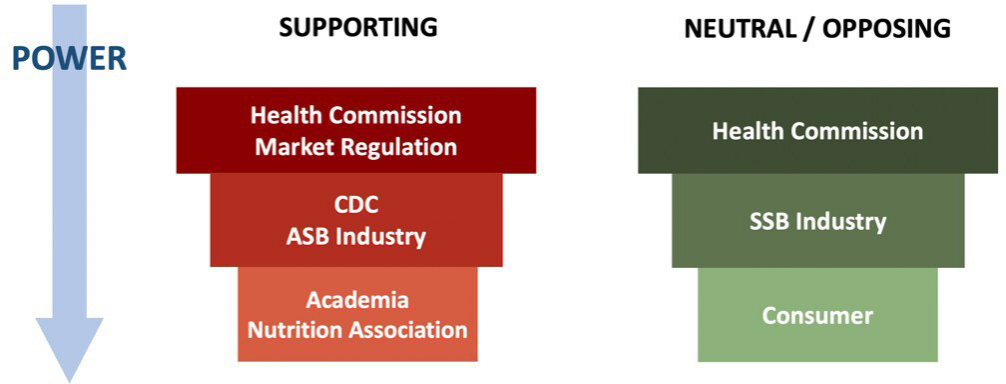
Potential key alliances of included stakeholders for SSB policy for children and adolescents in China. ASB, artificially sweetened beverage; SSB, sugar-sweetened beverage

From reviewing the literature on SSB policy analysis,^45^ four main groups of stakeholders were identified, namely, policymakers, nutrition experts, industry and consumers. Based on literature review results and expert consultation, we approach the following parties of key stakeholders as the interviewees, including policymakers (health commission, CDC and market regulation bureau), nutrition experts (academia and nutrition association), industry stakeholders (R&D and marketing managers from SSB and ASB companies) and consumers (primary caregivers of children and adolescents aged 6–17) (figure 1).

### Stakeholder analysis from qualitative interviews and discussions

Stakeholder analysis integrates insights from qualitative interviews and FGDs. These findings are presented thematically. Results are structured in two main parts: (i) stakeholders’ knowledge and perceptions of SSBs and related policies and (ii) perceived barriers and facilitators for adopting such policies in China. Within each theme, perspectives were compared across the four stakeholder groups to highlight areas of consensus and divergence.

This study included 9 policymakers, 2 nutrition experts, 5 industry stakeholders and 11 consumers. All qualitative participants provided input on their knowledge and perceived importance of SSB policies. Demographic characteristics of the included consumers are listed in online supplemental appendix 1A. The potential key alliance of included stakeholders is shown in figure 1. Stakeholders’ roles, work experience, city, involvement, interest, power and position in implementing SSB policies in China are presented in tables2  3.

**Table 2:** Involvement, interest, power and position of key informants and consumers (caregivers of children and adolescents)

Category	Stakeholders	Study site	Work experience	Interest*	Power†	Position‡
Policymakers	Decision-maker who is in charge of local standard formulation from provincial level health commission	Chongqing	Health and nutrition standard	High	High	Supportive
		Shanghai	Nutrition standard	High	High	Opposed
	Technical officer who is responsible for executing health decisions from the provincial-level CDC	Chongqing	Food and water surveillance	Medium	Medium	Supportive
		Chongqing	Child health	High	Medium	Supportive
		Chongqing	NCD prevention and control	High	Medium	Supportive
		Chongqing	Food and nutrition	High	Medium	Supportive
		Shanghai	Nutrition and epidemiology	High	Medium	Supportive
	Decision-maker who is in charge of market supervision and local standard formation from the municipal-level market regulation bureau	Chongqing	Standard, inspection of food and medical devices	Medium to high	High	Supportive
	Decision-maker who is in charge of market supervision and local standard formation from the provincial-level market regulation bureau	Shanghai	Standard, inspection of food safety	Medium to high	High	Supportive
Nutrition experts	Professor in nutrition from academia	Shanghai	Nutrition	Medium	Low	Supportive
	Nutrition expert from a provincial-level nutrition association	Shanghai	Nutrition	Medium	Low	Supportive
Consumers	Primary caregivers of children and adolescents	Chongqing, Shanghai	N/A	Low to medium	Low	Neutral

* Interest: stakeholders’ interest in introducing sugar-sweetened beverage (SSB) policies in China.

† Power: stakeholder’s decision-making power.

‡ Position: whether a stakeholder supports or opposes the SSB policy.

CDC, Centre for Disease Prevention and Control; N/A, not applicable; NCD, non-communicable disease.

**Table 3:** Involvement, interest, power and position of industry stakeholders

Category	Stakeholders	Product type*	Work experience	Interest†	Power‡	Position§
Industry	Department managers from the SSB industry	ASB, SSB	Research and development	High	Medium	Neutral
		ASB, SSB	Marketing	High	Medium	Opposed
		ASB	Marketing	Medium	Medium	Supportive
		SSB	Research and development	High	Medium	Neutral
		ASB, SSB	Research and development	High	Medium	Neutral

* Product type: this indicates the types of beverages for sale from the approached industry, which could be SSBs, ASBs or both.

† Interest: stakeholders’ interest in introducing SSB policies in China.

‡ Power: stakeholder’s decision-making power.

§ Position: whether a stakeholder supports or opposes the SSB policy.

ASB, artificially sweetened beverages; SSB, sugar-sweetened beverages.

#### Knowledge and perception of sugar-sweetened beverages and sugar-sweetened beverage policies for children and adolescents in China

##### Stakeholders’ knowledge of sugar-sweetened beverages and sugar-sweetened beverage policies for children and adolescents in China

All interviewed stakeholders acknowledged a growing trend of SSB consumption among children and adolescents in China, and they attributed this trend to the appealing taste of these beverages and the improved economic status and affordability of SSBs. Besides, health commission officers, CDC technical officers, academia and nutrition association experts also expressed concerns about the lack of nutrition knowledge among children and adolescents, as well as their primary caregivers.

Our economy has grown, people’s standard of living has improved and goods are abundant. Imagine 20 years ago, you may have wanted to drink SSBs, but you could not have them. CDC technical officer, key informant interview (Chongqing)

Most interviewed consumers considered beverages with a sweet taste as SSBs. Only one mother from Shanghai differentiated ASBs from SSBs based on the use of artificial sweeteners. Consumers also showed knowledge of the health risks associated with excessive SSB consumption. However, regarding the recommended intake amount, consumers showed unclear understanding. Additionally, caregivers mentioned the social aspect of SSB consumption, as their children often choose to have SSBs when meeting friends or engaging in leisure activities due to their taste and convenience.

When he sees others drinking SSBs, he will join them, and I will buy (SSBs)…you cannot let him be the left-out one. Caregiver, FGD (Shanghai)

Most key informants, except one market regulation officer from Chongqing, knew the policy types and implementation criteria of both international SSB policies and the domestic SSB policy pilot in Shenzhen, China. However, only one market regulation officer from Shanghai was familiar with international and domestic pilot SSB pilots. Conversely, all industry manufacturers interviewed understood international and domestic SSB policy specifics such as restricted beverage types, tax details and label design. Interestingly, among all the caregivers interviewed, only two from Shanghai had heard of international SSB taxes, and none of them noticed the Shenzhen pilot.

##### Perceived importance of sugar-sweetened beverage policies for children and adolescents in China

Most key informants supported the implementation of SSB policies in China. They believed these policies could effectively restrict SSB consumption among children and adolescents and improve health outcomes through consumer education and industry innovation. All key informants, except decision-makers from market regulation, were against the SSB tax, as they perceived the low effectiveness of small levy amounts and low public acceptance. They agreed that the most essential and appropriate way to control SSB consumption is through health education (eg, school programmes, public service announcements and healthy eating campaigns). Meanwhile, policymakers from market regulation believed that stricter policies, for example, taxes and mandatory FOP labelling, would be more effective, but they acknowledged the challenge that these policies would require national legislation to settle. Market regulation officers were also against marketing restrictions because of concerns about market fairness.

Tax, I think it may ultimately come down to the consumer end, and people’s purchasing power is still quite good now. I don’t think the effect of the SSB tax will be very good. Decision-maker from the health sector, key informant interview (Chongqing)

It would be good to broadcast a public service announcement, which connects to consumer education. Decision-maker from the marketing sector, key informant interview (Chongqing)

Industry stakeholders shared neutral opinions regarding SSB policies. They wished the SSB policies could guide consumers to make healthy choices instead of eliminating consumers’ freedom to choose. Furthermore, although ASB companies strongly supported SSB policies, other manufacturers brought up their challenges of substituting sugar with artificial sweeteners, including the unpleasant mouthfeel of artificial sweeteners, fear of losing consumers and challenges in innovation.

Technical challenge we face: can artificially-sweetened food taste as good or better than sugar-sweetened food?… Consumers also need education, and (SSB) policy is also a tough way to educate them…policies should guide consumers to make positive choices. Beverages R&D manager, in-depth interview

The interviewed consumers held mixed opinions. Some consumers believed that policy interventions could effectively control children’s SSB consumption, considering the challenges they face as caregivers in educating their children. On the contrary, some consumers perceived SSB policies as governmental interference in personal preferences, such as the SSB tax adding a financial burden without providing substantial benefits. Besides, caregivers leaned towards colour-coded FOP labels since their young children could easily understand.

FOP labels sound quite well. They can clearly tell the consumer which ones are high in sugar. It’s easier for me and my kid to choose. Caregiver, FGD (Chongqing)

I think the only way to is to have a policy that reformulates the beverages. For us consumers, it’s hard to control our own children. Caregiver, FGD (Shanghai)

If I really want to have a treat, do I really care about the calories? If all of them have a warning label, I have nothing to choose. Caregiver, FGD (Shanghai)

**Figure 2:**
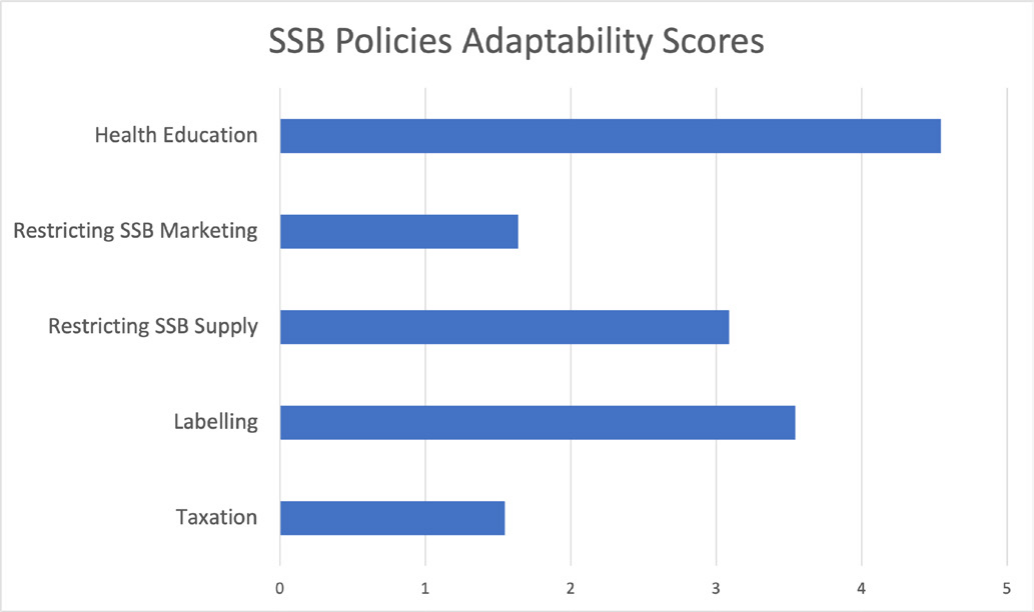
SSB policies’ adaptability scores. SSB, sugar-sweetened beverage

All key informants (n=11) were invited to rank the five categories of policies based on their adaptability if implemented in China. The most adaptable policy type was scored 5, and the least adaptable one was scored 1. The result of the ranking is shown in figure 2. Health education was voted as the most adaptable policy, followed by labelling. The least favoured policy type was taxation.

### Perceived barriers and facilitators for adopting sugar-sweetened beverage policies for children and adolescents in China

#### Legislation and standards

All key informants and industry stakeholders emphasised the need for a well-defined and comprehensive national standard to facilitate the implementation of SSB policies in China, regardless of the specific type of policy introduced. A CDC officer and a market regulation officer from Chongqing mentioned that standard establishment would outline specific requirements for the policies, including SSB definition, product types, sugar content definition and labelling requirements. Moreover, standards would encourage manufacturers to follow. According to the CDC, health commission and market regulation officers, coercive standards could encourage industry reformulation with clear requirements. Industry stakeholders also wish policymakers would understand their production difficulties and offer guidance through standard documents.

If the policy goes further, I suggest the standard (of nutrition labelling) can be more detailed. That is to say, including what kinds of added sugar (are used in the beverage), etc. This will give more detailed rules, so people (industries) also have a law to follow. CDC technical officer, key informant interview (Chongqing)

If the government provides clear policies, we will follow. Beverages R&D manager, in-depth interview

Furthermore, the health commission, CDC and market regulation stakeholders proposed that the current ‘National Food Safety Standard - Standard for nutrition labelling of prepackaged foods (GB 28 050–2011)’ could be revised to better align with the SSB restrictive policies, because the existing standard already includes ‘no-sugar’ or ‘low-sugar’ claims.

The current standard includes a definition of low sugar and sugar-free claims. If we want to go further (to implement SSB policies), I suggest refining this standard, adding more details. CDC technical officer, key informant interview (Chongqing)

#### Step-by-step policy

Except for consumers, all stakeholders reached a consensus on gradually introducing SSB policies in China, and the reasons are as follows. First, a gradual introduction allows the public to gain health knowledge, enabling them to adapt to the policies more effectively. Public health experts from the CDC, health commission, academia and nutrition association claimed the importance of consumers gaining health knowledge and awareness related to SSBs before full implementation. Second, market regulation officers mentioned that the industry will benefit from the gradual adoption of SSB policies. Third, the step-by-step delivery of policies will help different regions in China gradually adapt to SSB policies. However, two health sector key informants from Shanghai oppose nationwide implementation due to varying nutritional burdens and financial backgrounds across China. Therefore, a gradual implementation, adapting to local consequences, is considered helpful.

Different regions are facing different nutrition burdens…some remote regions still live off nutrition supplement subsidies, and they need sugar, while the obesity prevalence in Shanghai is high. Decision-maker from health sector, key informant interview (Shanghai)

#### Industry resistance and consumer acceptance

Policymakers highlighted the collective resistance from industries and industry associations (eg, beverage associations) as a barrier to implementing SSB policies in China. According to one market regulation officer and one CDC officer specialised in nutrition, establishing new standards and laws would require consensus between the government and the industry, and opposition from the industry would challenge the law from being in place.

In addition to our standardization committee of the State Administration of Market Supervision, we also invite many manufacturers to participate in forming standards. What if there are different opinions? For example, if my product is high in sugar, I will raise the (sugar content definition) standard to a very high level, for example, 10% is called high sugar content. It will be very difficult for us. Decision-maker from the marketing sector, key informant interview (Chongqing)

Several consumers displayed indifferent attitudes when asked about their views on introducing SSB policies in China. One health commission officer and one market regulation officer emphasised the importance of individual freedom. In addition, stakeholders from academia and industry argued that controlling SSBs may lead consumers to purchase other sweet products, and this substitution effect would not improve health outcomes.

(SSB policies) need to be accepted by the industry and the society to push further. Professor in nutrition, key informant interview (Shanghai)

Be aware of things that can be substitutes (of SSBs), like desserts. Decision-maker from health sector, key informant interview (Shanghai)

## Discussion

The result of this study showed that all interviewed stakeholders acknowledged a growing trend of SSB consumption among children and adolescents in China, as confirmed by previous literature about SSB intake in China.^16 46^ Most stakeholders claimed the increasing obesity prevalence is linked to rising SSB consumption, which aligns with the literature findings on China’s rising rates of overweight and obesity.^7 47^ As shown in figure 1, stakeholders differed in their support: health authorities, academia and nutrition associations tended to support SSB policies, whereas industry actors and some consumer representatives expressed opposition. Figure 2 further illustrates that policymakers and experts perceived certain measures, such as health education and labelling, as more adaptable, whereas taxation and marketing restrictions were seen as less feasible and effective. These findings provide a structured view of both stakeholder positions and policy implementation potentials.

The Chinese government has noticed that high SSB consumption would lead to rising obesity prevalence. However, this study revealed insufficient awareness among interviewed policymakers. Interviewed policymakers in Shanghai showed a better understanding of SSB policies compared with their peers in Chongqing, possibly due to the greater prevalence of obesity and overweight in Shanghai. Policy action-wise, Shanghai published the ‘Shanghai Nutrition Act’ and ‘Healthy Shanghai Plan’ and sought public opinions on these labels for policy preparation.^48^ In contrast, Chongqing’s policy actions are primarily at the advocacy level. This finding indicates the need for awareness-raising for policymakers, especially in less developed regions. Furthermore, international literature indicates that strong political will and governance from a collaborative government play an important role in implementing SSB policies.^49 50^ Based on the policy reviews, there is a positive political will from the Chinese government to promote healthy eating^19^, which may explain the industry’s neutral attitudes toward SSB policies, as they were alert to the opening of opposing governmental actions. Moreover, there are available resources for SSB policy introduction in China, which can be well utilised, since LMIC evidence suggested.^51^ The national standard for nutrition labelling of prepackaged foods (GB 28 050–2011) included requirements on sugar-free and low-sugar-content claims. Additionally, health education has been a part of the school curriculum for primary and secondary schools, although with less focus on nutrition.^52^ By building on these existing fundamentals, China can implement SSB policies more efficiently.

Regarding consumers, low health awareness and literacy pose significant challenges to implementing SSB policies. Interviewed policymakers in both municipalities were concerned about public acceptance owing to their lack of nutrition knowledge, potentially causing misunderstandings of the government’s decision, thus hindering policy implementation. In particular, consumers interviewed in this study are from urban cities, and the rural population’s nutrition awareness would be worse. A study suggests that insufficient nutrition education in schools contributes to low nutrition literacy among Chinese children.^52^ Domestic evidence showed that improving consumer awareness would enhance better acceptability of the SSB tax.^21^ International experience from Mexico also recognised the contribution of society organisations (eg, ‘The Nutrition Health Alliance’) to the successful implementation of SSB taxation owing to their help in raising public support.^53^ Thus, if China implements SSB policies, these measures would help enhance health literacy to improve public understanding and support.

According to our interview results, industry resistance is also identified as a major hindrance to enacting SSB policies in China. As figure 1 shows, the SSB industry is one of the strongest opponents of SSB policies, which reinforces the interpretation of industry resistance as a key barrier. The industry is aware of potential profit decline, innovation difficulties, rising reformulation costs and the fear of losing consumers. This finding is consistent with qualitative research among Dutch stakeholders, which highlights industries’ common concern about negative economic consequences.^49^ Thus, implementing an incremental, graded SSB policy would also foster industry innovation and reformulation and facilitate industry transformation.^50^ Introducing the policies step by step allows more time for the industry to adapt. However, challenges from industries also create obstacles for LMICs to adapt SSB policies. For instance, the industry’s collaborative policy opposition is promoted through step-by-step voluntary approaches as a tactic to delay the regulatory action. For example, industry groups from South Africa have been lobbying for years. They requested more time to prepare, and additional procedural requirements were used to slow down the adoption of the SSB tax.^54 55^ Furthermore, they also delayed the implementation of the SSB tax with the argument that ‘individuals should be responsible for their own SSB consumption rather than governments’,^50 56^ and the interviewed manufacturers in this study have also expressed similar attitudes. This shift of focus to individual responsibility rather than regulatory actions resonates with the strategies that major beverage manufacturers used in other contexts. For instance, Coca-Cola’s influence in China through advocating the importance of physical activity has distracted attention from SSB consumption as a key factor towards obesity.^57^ Other companies have also been setting up research institutes to generate evidence that avoids addressing the importance of nutrition and dietary health.^58^ Hence, international evidence suggested limiting the industry’s involvement in policymaking to minimise interference.^50^ The powerful and aggregated influence of industries can shape scientific discourse or even partner up with government agencies to promote strategies that prioritise their business interests.

Finally, more evidence in the Chinese context is needed to support SSB policy development in China. Studies on consumer responses are essential because SSBs are inexpensive and abundant in China and different from tobacco and alcohol.^18^ ^50^ Chinese evidence suggests the feasibility of an SSB tax in urban China owing to SSB’s high price elasticity and good public acceptance of the SSB tax,^21 59^ which contradicts our finding that urban consumers’ demand will not be affected by the price rise. Similar inconsistency regarding the effectiveness of SSB tax and marketing regulations has also been reported in other literature. In the Netherlands, academic experts and trade association stakeholders also doubted the effectiveness of SSB tax in reducing consumption, as they perceived purchasing behaviour to be shaped by various reasons other than price.^49^ In Iran, marketing and media stakeholders considered ‘low participation in enforcing the ban’, whereas key barriers were seen in conflicts of interest and insufficient penalties, suggesting limited effectiveness of potential marketing regulations through media channels.^60^

These inconsistencies of results may reflect differences in the study populations. Empirical household demand modelling from multiple high-income and middle-income countries shows that SSB purchases are highly price-responsive, particularly among low-income populations.^61^ However, the interviewees in this study were recruited from one of China’s most developed metropolitan areas (Shanghai) and the most developed urban district in a less developed city (Chongqing). The site selection may have introduced bias on higher-income urban populations, who are typically less price-sensitive. Thus, comprehensive evidence of the effectiveness of SSB policies in China is lacking, particularly in rural regions.^59 62 63^

Moreover, recent empirical research in China indicates that although SSB purchase declines, consumers would choose to purchase other sweet pastries and confectionery instead, leading to an overall increase of 2.57% in total caloric intake nationwide.^64^ This finding may undermine the potential effectiveness of SSB tax in reducing obesity prevalence. Further research on substitution effects, including shifts of consumer purchase on other sweet confectionery and untaxed beverages, would also be important to understand consumer behaviour.

In addition, Chinese researchers studied the association between near-campus SSB stores and obesity in school children, indicating that off-campus availability of SSBs could demonstrate a higher obesity risk.^65^ Such evidence suggested the potential feasibility of controlling SSB supply. However, less evidence is available for the feasibility of other SSB policies (eg, labels and marketing restrictions).

Besides, the evaluation of policy implementation would provide rich evidence. The involvement of domestic academia throughout policy evaluation is recommended because of accurate and cost-effective assessments in resource-limited settings.^50^ A clear evaluation plan with short-term, medium-term and long-term impact measurements would also provide valuable real-world evidence about policy delivery.

### Policy implications

Based on the findings above, China could launch a series of steps to introduce SSB policies. First, establishing strategies to enhance health literacy and nutrition awareness among both policymakers and consumers. Disparities in awareness still exist among policymakers and are uneven across the country. For informing policymakers, professional conferences, academia and health professional associations are good resources to promote awareness of restricting SSB intake. Besides, health education for consumers can be conducted through school education, national basic healthcare and medical service, and public service announcements. SSB policies, such as supply restrictions and health warning labels, are also ways to deliver public education. Figure 2 also emphasises that health education and labelling rated by stakeholders is the most adaptable policy option, suggesting that these could serve as entry points for initial implementation. Additionally, existing policy resources can be used for SSB policy development. Campus restriction of SSB could continue, given that it has achieved positive results and public support. Health warning labels on shelves could also be promoted at retail facilities. Then, the National Nutrition Plan (2017–2030) included specific standards for establishing ‘Nutrition Awarded Schools and Cafeterias’; restricting SSB supply in these facilities could be another criterion to encourage facilities to apply for this honorary title.

### Research implications

For policies requiring national legislation reforms (eg, taxation, FOP labels and marketing restrictions), more studies and pilot trials could be conducted to analyse their adaptability and scalability in China. Moreover, more evidence is needed to facilitate the nationwide implementation of SSB policies. Before researching further on implementation evidence, formative research can be conducted to study the community of interest comprehensively, considering their perspectives on SSB health issues. Once sufficient evidence is available to support policymaking, multiple policies targeting different aspects (eg, health education, SSB supply restrictions, health warnings and marketing restrictions) should be combined to foster a supportive niche for consumers to adopt healthier SSB consumption habits. Finally, a policy evaluation plan should be developed with short-term, medium-term and long-term goals and outcomes throughout the policy design and implementation period. Existing indicators for outcomes can be used, or new indicators can be designed to monitor policy implementation when applicable. In addition, academia and third-party evaluation teams are recommended to be involved in policy monitoring and effectiveness evaluation.

### Study limitations

This study has several limitations that could be addressed in future research. First, education sector stakeholders and children and adolescents themselves were not interviewed. Second, most evidence collected in this study was from urban regions, where the SSB supply could be different in rural settings. Additionally, the rural population with lower nutrition literacy may potentially lead to different consumption patterns. Third, only packaged SSBs were considered for this study, although handmade SSBs are popular in China among children, which should be considered in SSB policies. Moreover, only one male caregiver was recruited, as the requirement was being the primary caregiver of children, thus might reflect sex bias. Finally, the study was conducted during the COVID-19 pandemic with travel restrictions, so the data collection procedure from the two study sites was not identical, which might reveal differences in the information collected. Pandemic-related shifts in consumption habits, such as increased at-home beverage intake and different purchasing behaviour, may also mean that findings could differ if the study were repeated in a post-pandemic context.

## Conclusions

This study provides preliminary evidence on adopting SSB policies for future research to evaluate the effectiveness of these proposed political strategies in China. SSB intake is rising among Chinese children and adolescents, yet national SSB policies are absent because of policymakers’ insufficient awareness and lack of national standards. To address this, it is crucial to prioritise strategies that enhance health knowledge and awareness among both policymakers and consumers. This foundational step will help build a supportive environment, equipping for the future introduction of comprehensive national SSB policies, such as taxation and FOP labelling. By fostering a better understanding of the health risks associated with SSBs, these efforts can contribute to more effective and sustainable policy outcomes.

## Supplementary material

10.1136/bmjopen-2025-098746 online supplemental appendix 1

## Data Availability

Data sharing is not applicable as no datasets were generated and/or analysed for this study. Data are available upon reasonable request.
