# Supplementary material for: Developing policy on sugar-sweetened beverages for children and adolescents in China: a qualitative study of stakeholder views and perceptions
Source: BMJ Open. 2025 Dec 12;15(12):e098746. doi: 10.1136/bmjopen-2025-098746 (PMC12706188; doi:10.1136/bmjopen-2025-098746)
Supplement: online supplemental appendix 1 [file bmjopen-15-12-s001.docx]

Appendix A. Demographic characteristics of the interviewee

Table A.1: Demographic characteristics of interviewed consumers

| **Variables** |  | Total | Children  (Age 6-11) | Adolescents  (Age 12-17) |
| --- | --- | --- | --- | --- |
|  |  | (n=21) | (n=10) | (n=11) |
| **Primary Caregiver Role** | Mother | 18 | 9 | 9 |
|  | Father | 1 | 1 | 0 |
|  | Grandmother | 1 | 0 | 1 |
|  | Aunt | 1 | 0 | 1 |
| **Age** | 30-39 | 6 | 5 | 1 |
|  | 40-49 | 11 | 2 | 9 |
|  | 50 or above | 4 | 3 | 1 |

Appendix B. Interview Guides

Appendix B.1: Interview guide for government officers and nutrition experts (English version)

| **Topics** | **Prompts** |
| --- | --- |
| Opinion on introducing an SSB restriction policy in China | Support or against, type of policy |
| Advantages and disadvantages of an SSB restriction policy (tax, front-of-pack label, warning label on shelves, restriction in school, education) | Purchases and consumption of SSB, health-related outcomes, product reformulation, government intervention, socioeconomic equality, economic consequences |
| Support and opposition of stakeholders | The public, health and consumer organizations, health professional associations, the food and beverage industry, academics, policymakers, government, international stakeholders |
| The feasibility of the decision to implement an SSB restriction policy in China | Choice of policy, support of stakeholders, communication between stakeholders, scientific evidence, successes in other countries, use of revenues (tax), administration, legal framework, type of product restricted |
| Facilitators and barriers of the potential future implementation of an SSB policy in China (at city, province and country level) | Stakeholder perspectives, vertical and horizontal communication, public acceptability, scalability |
| Recommendations for the design of an SSB restriction policy if introduced in the Chinese context | Definition of SSB in China, tax rate, use of tax revenues, type of label (recommended or compulsory), school restriction |
| Recommendations for alternative measures to reduce overweight and obesity in China |  |

Appendix B.2: Interview guide for industry stakeholders (English version)

| **Topics** | **Prompts** |
| --- | --- |
| China’s SSB market development | Future trend, popular products, consumer choices |
| Opinion on introducing an SSB restriction policy in China | Support or against, type of policy |
| Advantages and disadvantages of an SSB restriction policy (tax, front-of-pack label, warning label on shelves, restriction in school, education) | Purchases and consumption of SSB, health-related outcomes, product reformulation, government intervention, socioeconomic equality, economic consequences |
| Support and opposition of stakeholders | The public, health and consumer organizations, health professional associations, the food and beverage industry, academics, policymakers, government, international stakeholders |
| The feasibility of the decision to implement an SSB restriction policy in China | Choice of policy, support of stakeholders, communication between stakeholders, scientific evidence, successes in other countries, use of revenues (tax), administration, legal framework, type of product restricted |
| Response strategies if SSB policy is in place in China | Innovation directions, previous work and current actions, communication between stakeholders, international experience |
| Recommendations for the design of an SSB restriction policy if introduced in the Chinese context | Definition of SSB in China, tax rate, use of tax revenues, type of label (recommended or compulsory), school restriction |
| Industry engagement in public health programs and policies | Missions and goals, precious work, future actions, channel of communication, multistakeholder collaboration |

Appendix B.3: Focus Group Discussion Guide for Consumers (English version)

| **Topics** | **Prompts** |
| --- | --- |
| Current SSB consumption habit (without SSB policy) | Consumption amount and frequency now, change in habit if introducing an SSB policy, purchase of SSB, knowledge of SSB health risks |
| Opinion on introducing an SSB restriction policy in China | Support or against, type of policy, acceptability |
| Whether SSB consumption habits would change with SSB policy | Change in consumption amount and frequency, change choice of beverages |
| Advantages and disadvantages of an SSB restriction policy (tax, front-of-pack label, warning label on shelves, restriction in school, education) | Purchases and consumption of SSB, health-related outcomes, product reformulation, government intervention, socioeconomic equality, economic consequences |
| Recommendations for the design of an SSB restriction policy if introduced in the Chinese context | Acceptable tax rate, type of label (recommended or compulsory), school restriction |
| Recommendations for alternative measures to reduce overweight and obesity in China |  |

# Appendix C. Informed Consent Script

***English Version***

**Oral Informed Consent Script**

Thank you for your interest and time in talking with me.

In this study, our team wants to investigate the adaptability and scalability of restriction policies on sugar-sweetened beverages in China. If you choose to be a part of this project, I will have an interview with you, where I will ask a range of questions about your opinions on the introduction of this policy in China, and what type of policy would better adapt and scale up in China. We can talk for as long as you like, but I expect our conversation will take about an hour. I would also like your permission to let me audio-record our conversation so that I can focus on our conversation rather than taking notes. With your permission, I would like to make an audio recording of our discussion to make sure I’m getting an accurate record of the interview. Do you give your permission for me to interview and audio record you?

Although I will include your identity in my research notes, your identity or personal information will not be disclosed in any publication of the study. Instead, I will use a fake name or report my results in groups and averages so no one can be recognized. Although collected data may be made public or used for future research purposes, your identity will always remain confidential. I will store the research data safely and confidentially in encrypted storage provided by DKU. Only people in my research team could have access to the data.

You can ask me any questions at any stage of our interview, and you can skip any question that you do not want to answer. You can also stop our interview at any time, for any reason. If you do not want to be recorded, that is okay. Whatever you decide to do is fine, I fully respect your decision.

Please let me know if you have any questions for me at this time. If you have any other complaints or concerns later, you can also contact me through my email.

I fully respect your opinion of whether you will participate. Do you have any questions? Are you happy to take part?

Ok, thank you, let’s start the interview.

# APPENDIX D. Definition of SSB policies discussed in this article

1. **Taxation** applies a levy onto SSB products.
2. **Labelling** involves front-of-package (FOP) labels and shelf labels, in which FOP labels are placed on the package of SSB products, including warning labels (e.g. ‘High in” labels from Mexico), rating labels (e.g. Health Star Rating from Australia and New Zealand) and color-coded labels (e.g. Nutri-Grade from Singapore) on the package of SSB products. Shelf labels are placed on store shelves where SSB products are displayed, these labels may include health warnings or health advising captions (e.g. Health warning labels from Shenzhen, China).
3. **Restricting SSB supplies** includes various methods such as limiting the availability of SSBs in specific locations such as schools, governmental institutions, and hospitals. It also includes increasing the availability of non-SSB products in retail stores (e.g. placing more plain water in the beverage sector of stores, and promoting non-SSB products in more prominent locations at retail facilities).
4. **Restricting SSB marketing and advertising** refers to banning or limiting the advertisement of SSB products through media channels, such as banning SSB advertisements on children’s television channels.
5. **Health education** entails activities that promote awareness of the harms associated with SSB consumption and encourage healthy eating. For instance, providing health education classes in schools, and creating public service announcements for the public.

**Appendix E. COREQ checklist**

Consolidated criteria for reporting qualitative studies (COREQ): 32-item checklist

Developed from:

Tong A, Sainsbury P, Craig J. Consolidated criteria for reporting qualitative research (COREQ): a 32-item checklist for interviews and focus groups. International Journal for Quality in Health Care. 2007. Volume 19, Number 6: pp. 349 – 357

| **Item No** | **Guide Questions/Description** | **Remarks** |  |  |
| --- | --- | --- | --- | --- |
| **Domain 1: Research team and reflexivity** | | |  |  |
| **Personal Characteristics** | | |  |  |
| 1. Interviewer/ facilitator | Which author/s conducted the interview or focus group? | Noted in Page 9 Methods – Qualitative Interviews – Data Collection. YS conducted the interviews. |  |  |
| 2. Credentials | What were the researcher’s credentials? E.g., PhD, MD | YS holds a BSc, and was pursuing her MSc degree at the time of this study. JZ holds a PhD. JW holds an MPH. QS holds an MBBS. QL holds a PhD. |  |  |
| 3. Occupation | What was their occupation at the time of the study? | YS was an MSc student. JZ, JW and QS were CDC technical officers. QL was an assistant professor and was the supervisor of YS. |  |  |
| 4. Gender | Was the researcher male or female? | Female. |  |  |
| 5. Experience and training | What experience or training did the researcher have? | Noted in Page 9 Methods – Qualitative Interviews – Data Collection. YS was trained in qualitative methods. |  |  |
| **Relationship with participants** | | |  |  |
| 6. Relationship established | Was a relationship established prior to study commencement? | YS, JZ, JW and QS organized interview schedules with the interviewees. Besides this, YS did not have direct connection with interviewees before the interviews. |  |  |
| 7. Participant knowledge of the interviewer | What did the participants know about the researcher? e.g. personal goals, reasons for doing the research? | During the oral informed consent prior to each interview, YS introduced her role and the reason to conduct this study. |  |  |
| 8. Interviewer characteristics | What characteristics were reported about the interviewer/facilitator? e.g. Bias, assumptions, reasons and interests in the research topic | This study was conducted as a component of YS’s Master’s thesis. This information was disclosed to all interviewees during the oral informed consent procedure before every interview. |  |  |
| **Domain 2: Study design** | | |  |  |
| **Theoretical framework** | | |  |  |
| 9. Methodological orientation and Theory | What methodological orientation was stated to underpin the study? e.g. grounded theory, discourse analysis, ethnography, phenomenology, content analysis | Noted in Page 7 Methods – Study Design. The study design referenced a theoretical framework “Analyzing and addressing governance in sector operations”. |  |  |
| **Participant selection** | | |  |  |
| 10. Sampling | How were participants selected? e.g., purposive, convenience, consecutive, snowball | Noted in Page 9 Methods – Qualitative Interviews – Data Collection. Policymakers, nutrition experts, industry stakeholders were recruited through purposive sampling. Consumers were recruited through convenience sampling. |  |  |
| 11. Method of approach | How were participants approached? e.g., face-to-face, telephone, mail, email | Noted in Page 9 Methods – Qualitative Interviews – Data Collection. Policymakers, nutrition experts, industry stakeholders were recruited through professional connections. Consumers were recruited through convenience sampling by community health centre workers. |  |  |
| 12. Sample size | How many participants were in the study? | Noted in Page 14 Results – 2. Stakeholder Mapping of SSB Policy Implementation in China. A total of 37 participants were included. |  |  |
| 13. Non-participation Setting | How many people refused to participate or dropped out? Reasons? | One industry stakeholder didn’t participant due to no response of invitation. |  |  |
| 14. Setting of data collection | Where was the data collected? e.g., home, clinic, workplace | Noted in Page 9 & 10 Methods – Qualitative Interview – Data Collection. Participants in Chongqing were interviewed through virtual meetings. Participants in Shanghai were interviewed in person either in their office (policymakers, nutrition experts, industry) or the community centre (consumers). |  |  |
| 15. Presence of nonparticipants | Was anyone else present besides the participants and researchers? | No, only the participants and the interviewer were present during interviews. |  |  |
| 16. Description of sample | What are the important characteristics of the sample? e.g. demographic data, date | Consumers (primary caregivers of children and adolescents) recruited were mostly female and as the role of mothers. Although the sampling didn’t tend to recruit solely female participants and the focus was primary caregivers that know their children’s SSB consumption habit, female and mothers are more commonly to be the primary caregivers. |  |  |
| **Data collection** | | |  | No |
| 17. Interview guide | Were questions, prompts, and guides provided by the authors? Was it pilot tested? | Noted in Page 9 Methods – Qualitative Interview – Data Collection. YS conducted mock interviews before the formal interviews with participants. The interviews were semi-structured and questions were delivered to participants from YS verbally. |  |  |
| 18. Repeat interviews | Were repeat interviews carried out? If yes, how many? | No repeat interviews were conducted. |  |  |
| 19. Audio/visual recording | Did the research use audio or visual recording to collect the data? | Noted in Page 10 Methods – Qualitative Interview – Data Collection. Most interviews were video recorded except one with a policymaker (detailed notes were taken during this interview). |  |  |
| 20. Field notes | Were field notes made during and/or after the interview or focus group? | Noted in Page 10 Methods – Qualitative Interview – Data Collection. One with a policymaker were recorded through taking detailed notes during this interview. |  |  |
| 21. Duration | What was the duration of the interviews or focus group? | Noted in Page 10 Methods – Qualitative Interview – Data Collection. Each interview lasted for 45 to 60 minutes |  |  |
| 22. Data saturation | Was data saturation discussed? | Noted in Page 11 Methods – Qualitative Interview – Quality Assurance. Quality and trustworthiness of the collected qualitative data interpretation were assured through triangulating findings. |  |  |
| 23. Transcripts returned | Were transcripts returned to participants for comment and/or correction? | No transcripts were returned to participants. |  |  |
| **Domain 3: analysis and findings** | | |  |  |
| **Data analysis** | | |  |  |
| 24. Number of data coders | How many data coders coded the data? | Noted in Page 10 Methods – Qualitative Interview – Data Analysis. One author (YS) familiarized the transcripts to identify key points for developing the coding scheme after discussing with the author who was most experienced in qualitative research among the team (QL). |  |  |
| 25. Description of the coding tree | Did the authors provide a description of the coding tree? | Noted in Page 10 Methods – Qualitative Interview – Data Analysis. |  |  |
| 26. Derivation of themes | Were themes identified in advance or derived from the data? | Noted in Page 10 Methods – Qualitative Interview – Data Analysis. Themes were developed from the data. |  |  |
| 27. Software | What software, if applicable, was used to manage the data? | Noted in Page 10 Methods – Qualitative Interview – Data Analysis. NVivo was used to manage the data. |  |  |
| 28. Participant checking | Did participants provide feedback on the findings? | No transcripts were returned to participants for feedback. |  |  |
| **Reporting** | | |  |  |
| 29. Quotations presented | Were participant quotations presented to illustrate the themes/findings? Was each quotation identified? e.g., participant number | Noted in Page 15 – 22 Results – 3. Stakeholder Analysis with identification. |  |  |
| 30. Data and findings consistent | Was there consistency between the data presented and the findings? | Yes. |  |  |
| 31. Clarity of major themes | Were major themes clearly presented in the findings? | Yes. |  |  |
| 32. Clarity of minor themes | Is there a description of diverse cases or a discussion of minor themes? | Yes. |  |  |
