# Supplementary material for: Developing policy on sugar-sweetened beverages for children and adolescents in China: a qualitative study of stakeholder views and perceptions
Source: BMJ Open. 2025 Dec 12;15(12):e098746. doi: 10.1136/bmjopen-2025-098746 (PMC12706188; doi:10.1136/bmjopen-2025-098746)
Supplement: Supplementary file 2 [file bmjopen-2025-098746.reviewer_comments.pdf]

PEER REVIEW HISTORY

BMJ Open publishes all reviews undertaken for accepted manuscripts. Reviewers are asked to complete a checklist review form and are provided with free text boxes to elaborate on their assessment. These free text comments are reproduced below.

ARTICLE DETAILS

Title (Provisional)

Developing policy on sugar-sweetened beverages for children and adolescents in China: a qualitative study of stakeholder views and perceptions

Authors

Suo, Yue; Zang, Jiajie; Wang, Jia; Shen, Qianqian; Long, Qian

---

VERSION 1 - REVIEW

**Reviewer**	**1**
**Name**	**Niknam, Mahdieh**
**Affiliation**	**Shaheed Beheshti University of Medical Sciences, Community Nutrition**
**Date**	**12-Mar-2025**
**COI**	**None**

Dear Editor,

Greetings!

I would like to extend my gratitude for the opportunity to review the manuscript submitted to BMJ Open. The study seeks to explore stakeholder perceptions regarding SSB policies for Chinese children and adolescents, as well as the facilitators and challenges of policy implementation. Overall, the manuscript addresses an important public health issue. It is well-written, and key sections such as the abstract, introduction, and methods are clearly described and aligned with the study's objectives. However, I have a few suggestions that could further enhance the manuscript and address certain areas for improvement:

1. In this study a theoretical framework entitled "Analyzing and addressing governance in sector operations', which involved following three steps – context analysis, mapping stakeholders and stakeholder analysis. Please considered a separate section in the method entitled "Conceptual framework" and defined this theory comprehensively.

2. The study was conducted in two Chinese municipalities, Shanghai and Chongqing; however, the rationale for selecting these locations was not provided. These areas appear to have diverse socioeconomic statuses (SES). If this was a factor, the study did not report or

discuss the differences in results observed between the two regions. as well as the differences of the

3. The discussion of the study should reflect how the main findings align with the study's objectives. The primary aim was to examine stakeholder perceptions of SSB policies for Chinese children and adolescents, along with the facilitators and barriers to policy implementation. However, the current statement does not fully capture the core findings. For example, the results presented in Figures 1 and 2 offer significant insights that warrant further exploration. Therefore, it is essential to revise the discussion to better emphasize and align with the study's main results and findings.

**Reviewer**	**2**
**Name**	**Forde, Hannah**
**Affiliation**	**University of Oxford, Nuffield Department of Primary Care Health Sciences**
**Date**	**15-Jul-2025**
**COI**	**None**

General comments

• The researchers have focused on an important policy area, in a context that is generally understudied, so this paper could make a valuable contribution of the field.

• The researchers have interviewed a range of key informants who are often hard to involve in research (e.g., members of the SSB industry), which is a key strength of this study.

• The manuscript could do with some reorganisation and clarification in places, to make clearer how the research was conducted and therefore, the value of resultant findings. In particular, the manuscript oscillates between presenting 3 stages of work or simply focusing on one (the interviews). I think it would be helpful to either only focus on the interview work, and just briefly summarise how the other two stages fed into it, or to more evenly describe the three stages throughout.

• Though I appreciated the inclusion of COREQ reporting, greater transparency about the methods is required in the main text to ensure your research can be fully understood and appraised.

Abstract

• The abstract is mostly well written and summarises the research clearly.

• The results section tends to focus mostly on the interview findings, rather than on the policies and literature reviewed e.g. is it possible to add numbers to the volume of literature and/or policies reviewed?

Highlights

• The tense of the highlights seems off – should it be more like e.g. The “analysing and addressing governance in sector operations” framework was adapted to…” etc.

Introduction

• Line 67: add references for SSB consumption growing worldwide. Might want to make clearer which ones it’s growing in vs those where it’s stable/reducing. Also helpful to bring the SSB definition before this sentence.

• Line 75: “The effect was mainly positive” – this could be reworded to be a little clearer (since I’m not sure soft drinks companies would be say the effect was positive!). Perhaps you could reference a systematic review of SSB taxes and briefly describe their impacts e.g., reduced purchases, changes in volume of sugar in drinks. That might mean you could also lose the specific example of Mexico (if you wanted to, though I appreciate that’s a good LMIC example).

• Line 98: rather than ‘proclaimed’ maybe announced or implemented would be more appropriate terms?

• Line 99: Since you’ve already laid out the health impacts of SSBs, not sure the sentence starting "Unrestricted SSB intake..." is needed.

• Line 100: Sentence starting “Considering China’s geography…” It’s not obvious to readers unfamiliar with China’s context why their geography/demographic structure/economic development might affect the feasibility of an SSB policy. Could you be more specific and/or expand on this with references?

• 101: Similarly, can you be more specific about the knowledge gap e.g. are there no studies? Or only a few that aren’t very relevant? Or something else?

• I think doing the above would help achieve a clearer justification for why you needed to speak with those particular stakeholders, and how this relates to your aims, which is currently a little vague.

• It would be helpful somewhere in the introduction to define what you consider SSB-related policies to be (e.g. are generally junk food advertising restrictions within scope?), both to add clarity in the intro but also to make clear what you’re focusing on throughout the rest of your study. I see that later on you have a detailed description of SSB policies within scope in Appendix D, which is helpful, but I think some kind of definition in the main paper earlier on would aid the reader.

• It would be sensible to reflect on how Coca-Cola's policy influence might also be restricting policy action on SSBs in China. See Susan Greenhalgh's work (here and/or in the discussion) https://www.bmj.com/content/364/bmj.k5050

Methods

• Line 109: I'm not sure that 'referenced' is the most specific term for how the theoretical framework was used in your study. Could you say something like 'underpinned' or 'directly informed' or something else? In the study design section, I think you could also refer to it being a qualitative study. Also, it's not clear why using this theoretical framework was particularly important in this context and/or what it offered over other comparable frameworks. Can you explain and add a further description for what the different stages are?

• I think a reference is missing for the GDP data on lines 115 and 117.

• Line 122: "Context analysis included policy review and literature review". Was anything else done for the context analysis? This sort of implies there was. If not, could rephrase to: "Context analysis consisted of policy review and literature review".

• Can you explain more about the process for conducting the policy review e.g., how did you define SSB-related policies, did you have key terms for searches, inclusion/exclusion criteria, restrictions by date, can you supply a full list of the international organizations you searched? The current description doesn't provide enough detail to make clear how the research was conducted.

• Similarly, there is insufficient description of the literature review process for it to be replicable. I suggest consulting with literature review reporting guidance (e.g. PRISMA), to check you've included all that might be expected for this type of work.

• If a light touch approach was taken to either the policy review and/or the literature review that would make addressing the two points above difficult, you should consider reporting this differently e.g. a rapid review of policy/literature to inform qualitative interviews. But I think addressing the above two points would be preferable.

• Line 134: "Policy metrics were used to determine the policy environment in China". Not clear what this means, what policy metrics are you referring to?

• Line 135: What did the expert consultation consist of?

• Public involvement: this sounds more like members of the public were recruited as fieldworkers to distribute recruitment information, rather than as public involvement. Can you explain more the impact of consumers (of SSBs?) holding this roles and/or what impact it had on your research? Also, I'm not sure this is the best location for this section, since you haven't introduced recruitment of interviewees yet.

• Line 144: Maybe first say who you did interviews with, and who you did focus groups with.

• Sentence starting line 146 would be more appropriate for the results section. Appendix A: I'm not sure if "role" is the best descriptor for the third column. Maybe "family role" or "primary caregiver role" or something else?

• Line 151: more description of procedure is required e.g. approached by email/phone/something else? What were your eligibility criteria for each group? What information was provided to each type of participant in advance of data collection e.g. participant information sheet? It's not clear when you switch from talking about consumers to primary care givers whether these are the same or a different group?

• Line 164: I'm not clear of what a focus group discussion interview is (sounds like two different methods)? Were the focus groups only for consumer groups? How long did focus groups last? If focus groups were indeed used, you should refer to them more clearly throughout, as at the moment I only see mentions of interview hereafter.

• Line 177: why was one interview not recorded?

• Line 180: how was the transcription achieved? By hand/AI/something else?

• Line 193: I'm not sure this needs a full section, could be condensed and subsumed into your analysis section.

• Line 181: can you explain a bit more about the stages of thematic content analysis? This will help the reader understand what structural codes are etc. Also, I think there's a typo (familiarisation with?).

• Overall the analysis section is well described.

• Sentence starting line 188: think this could go at the start of the methods section.

• Line 199: on what date was approval granted?

• It's not currently clear in your methods how stakeholder mapping was achieved – a description of methodological approach should be added.

• Note that I the interview guides imply the interviews were unstructured to semi-structured – if that's correct, it would be helpful to add that description to the

Results

• Signposting at the start of this section could help the reader navigate it e.g. it's divided into the parts to report…etc.

• Section 1.1: while relevant to your study, it's not obvious to the reader how this would be the product of your context analysis, since at the moment the description of methods focuses more on the policy actions (I think the signposting might help).

• Line 210: not clear what a P95 value is.

• Section 1.2: I think appendix d is a clearer articulation of the types of potential policy options. I wonder if this section could be presented in a more visually appealing way e.g. timeline of key policies, nationally and per location? Or a table?

• Section 2: I don't think the stakeholder mapping is currently very clear. Did you physically map and if so on what software? I think better description in the methods would help.

• Section 3: I think line 262 belongs in section 3.

• Line 269: how was interest/power/position assessed? Not currently clear.

• Lines 271 – 276 would be better suited to the methods.

• Section 3: How is this section organised? By theme or something else? Some signposting would help.

• Line 294 typo – the health the health

• Line 304 typo? Except instead of expect?

• Section 3.1.2. – as previously mentioned, the fact that you discuss support for SSB policies means it would be helpful to say up front how those policies were articulated to interviewees.

• Line 362 – that the interviews included rankings should be described in the methods, so that here you can simply present the results of the rankings. Also, it would be helpful to know what is meant by adaptability, as this wasn't obvious to me.

• Figure 2 – how were these scored? How were 0.5 points achieved? How many people responded to these questions?

Discussion

• You've made some good reflections on your work, but the discussion could do with some improvement in places. It's currently missing a few things I might expect to see in a discussion, you might want to considering structuring as follows: summary of key findings, strengths and limitations, comparison to existing literature, interpretation, policy implications. In particular, comparing your findings to other comparable research (e.g., qualitative research, not just literature identified in your review), might help interpret your findings in China's context specifically.

• At the moment, your discussion primarily reflects on your interview results, but your literature/policy review (and stakeholder mapping, though see comment above), were also key parts of your paper that should be reflected upon more clearly. That said your paragraph starting on line 449 does a better job of integrating the results (well done!).

• Sentence starting on line 459: I'm not sure that it's obvious why your second clause follows your first?

• The results present a lot of arguments often used by industry to slow the implementation of policies that might affect them, and I agree with your identification/reflection on this in the discussion. I think this could be expanded on further e.g. some might consider introducing step-by-step policies is also an industry tactic!

• Paragraph starting 496: you make some good points in this paragraph, but at the moment it's quite hard to follow as it spans a lot of different issues – consider reorganising and/or breaking into smaller paragraphs to aid the reader?

• Policy implications section: whilst I don't necessarily disagree with these suggested actions, it's not clear how these are all direct policy implications from your research. Suggest focusing only on those that are directly related to your research. I think this section could also be split into research at and policy implications, which at the moment are quite blended.

• Limitations: try to draw out more what the limitations of the study might mean for your research i.e. how does evidence mostly collected from urban regions affect the transferability of your findings? Were consumption habits different in COVID and thus you'd find something different if you repeated the study now? Etc

---

VERSION 1 - AUTHOR RESPONSE

Reviewer: 1
Ms. Mahdieh Niknam, Shaheed Beheshti University of Medical Sciences
Comments to the Authors:
Dear Editor,
Greetings!
I would like to extend my gratitude for the opportunity to review the manuscript submitted to BMJ Open. The study seeks to explore stakeholder perceptions regarding SSB policies for Chinese children and adolescents, as well as the facilitators and challenges of policy implementation. Overall, the manuscript addresses an important public health issue. It is well-written, and key sections such as the abstract, introduction, and methods are clearly described and aligned with the study's objectives. However, I have a few suggestions that could further enhance the manuscript and address certain areas for improvement:

1. In this study a theoretical framework entitled "Analyzing and addressing governance in sector operations', which involved following three steps – context analysis, mapping stakeholders and stakeholder analysis. Please considered a separate section in the method entitled "Conceptual framework" and defined this theory comprehensively.
Author's response: Thank you so much for your comments. I have added a separate section entitled "Conceptual framework" at the Method section (please see line 134-137).

2. The study was conducted in two Chinese municipalities, Shanghai and Chongqing; however, the rationale for selecting these locations was not provided. These areas appear to have diverse socioeconomic statuses (SES). If this was a factor, the study did not report or discuss the differences in results observed between the two regions. as well as the differences of the
Author's response: We have elaborated further on why we chose these two sites due to their disparities in geographical location and socioeconomic levels (line 146-152)

3. The discussion of the study should reflect how the main findings align with the study's objectives. The primary aim was to examine stakeholder perceptions of SSB policies for Chinese children and adolescents, along with the facilitators and barriers to policy implementation. However, the current statement does not

fully capture the core findings. For example, the results presented in Figures 1 and 2 offer significant insights that warrant further exploration. Therefore, it is essential to revise the discussion to better emphasize and align with the study's main results and findings.
Author's response: Thank you for pointing this out. We have revised the Discussion to explicitly reflect how the findings align with the study objectives and to integrate Figures 1 and 2 more clearly. Specifically – (1) At the end of the first paragraph of the discussion section (line 564-670), (2) a sentence that notes Figure 1 identified the SSB industry as one of the strongest opponents (line 708-710), (3) a sentence that indicates Figure 2's emphasis on health education and labelling being chosen by the most adaptable two policy types (line 780-783).

Reviewer: 2
Dr. Hannah Forde, University of Oxford
Comments to the Authors:
Abstract

• The results section tends to focus mostly on the interview findings, rather than on the policies and literature reviewed e.g. is it possible to add numbers to the volume of literature and/or policies reviewed?
Author's response: Thank you for the comments. We conducted a rapid review to inform qualitative interviews, thus the number of literature/policy reviews was not mentioned in the abstract section. While in the main body of text, we added descriptions of the primary terms we used for the literature search (line 207-209).

Highlights
• The tense of the highlights seems off – should it be more like e.g. The "analysing and addressing governance in sector operations" framework was adapted to…" etc.
Author's response: Thank you for your comments. The tenses have been corrected accordingly (line 38).

Commented [QL1]: Make it clearly about your response.

Introduction
• Line 67: add references for SSB consumption growing worldwide. Might want to make clearer which ones it's growing in vs those where it's stable/reducing. Also helpful to bring the SSB definition before this sentence.
Author's response: Reference has been added accordingly regarding growing SSB consumption worldwide. The definition of SSB has been placed as the first sentence of the introduction, followed by the increasing consumption sentence (line 65-83).

• Line 75: "The effect was mainly positive" – this could be reworded to be a little clearer (since I'm not sure soft drinks companies would be say the effect was positive!). Perhaps you could reference a systematic review of SSB taxes and briefly describe their impacts e.g., reduced purchases, changes in volume of sugar in drinks. That might mean you could also lose the specific example of Mexico (if you wanted to, though I appreciate that's a good LMIC example).
Author's response: We have added a reference of a systematic review of SSB policy impact on declined purchases, and tried to keep the example of Mexico to emphasize the effect of such policies in LMIC (line 82-84, reference number 13-14).

Commented [QL2]: Please add this reference number after updating the reference list. If you made any change in the text, please add the clarification here too.

• Line 98: rather than 'proclaimed' maybe announced or implemented would be more appropriate terms?
Author's response: I have changed "proclaimed" to "announced", which suits better (line 107).

• Line 99: Since you've already laid out the health impacts of SSBs, not sure the sentence starting "Unrestricted SSB intake..." is needed.
Author's response: Here, we would like to emphasize that no policy actions have been implemented to restrict/advise SSB consumption among children and adolescents, thus the SSB intake would be more "unrestricted" to Chinese children and adolescents (line 109).

• Line 100: Sentence starting "Considering China's geography…" It's not obvious to readers unfamiliar with China's context why their geography/demographic structure/economic development might affect the feasibility of an SSB policy. Could you be more specific and/or expand on this with references?

Author’s response: We have made changes accordingly to address that regional/geographical disparities and different levels of economic development in China might lead to different responses towards SSB policies (line 110-112, reference number 21).

• 101: Similarly, can you be more specific about the knowledge gap e.g. are there no studies? Or only a few that aren’t very relevant? Or something else?
Author’s response: We explained in more detail that currently there is a knowledge gap in assessing stakeholder perspectives of SSB policies in China.

• I think doing the above would help achieve a clearer justification for why you needed to speak with those particular stakeholders, and how this relates to your aims, which is currently a little vague.
Author’s response: Thank you so much for pointing out the issues listed above. We have also did some edits towards the study objective to make it more specific (line 114 – 117).

• It would be helpful somewhere in the introduction to define what you consider SSB-related policies to be (e.g. are generally junk food advertising restrictions within scope?), both to add clarity in the intro but also to make clear what you’re focusing on throughout the rest of your study. I see that later on you have a detailed description of SSB policies within scope in Appendix D, which is helpful, but I think some kind of definition in the main paper earlier on would aid the reader.
Author’s response: For this study, we would like to restrict SSB-related policies to policies directly targeting SSBs (although some of them might be targeting multiple junk food groups, such as high salt and high fat foods). In the introduction section, I brought up some of these policies in China because SSBs were a component of the policy announced, so they still count as SSB policies.
And a clearer mention of SSB policies was added in the introduction section accordingly (line 107-109).

• It would be sensible to reflect on how Coca-Cola’s policy influence might also be restricting policy action on SSBs in China. See Susan Greenhalgh’s work (here and/or in the discussion) https://www.bmj.com/content/364/bmj.k5050
Author’s response: Thank you for suggesting this source, I included this reference in the discussion section (line 730, reference number 57).

Methods
• Line 109: I’m not sure that ‘referenced’ is the most specific term for how the theoretical framework was used in your study. Could you say something like ‘underpinned’ or ‘directly informed’ or something else? In the study design section, I think you could also refer to it being a qualitative study. Also, it’s not clear why using this theoretical framework was particularly important in this context and/or what it offered over other comparable frameworks. Can you explain and add a further description for what the different stages are?
Author’s response: A more detailed introduction of the theoretical framework, plus the reason we used it, has been added to the appropriate section of this manuscript (as this framework is suitable for the study context when the evidence is limited). Your suggested wording has been applied as well. By using this framework, it guides me to analyze this complex policy issue with a more strategic approach, especially when there is limited evidence (line 138-143).

• I think a reference is missing for the GDP data on lines 115 and 117.
Author’s response: I added the reference for the GDP data (line 149 and 151).

• Line 122: “Context analysis included policy review and literature review”. Was anything else done for the context analysis? This sort of implies there was. If not, could rephrase to: “Context analysis consisted of policy review and literature review”.
Author’s response: Thank you for the suggestion of the wording. Context analysis only has two parts – policy review and literature review (line 155).

• Can you explain more about the process for conducting the policy review e.g., how did you define SSB-related policies, did you have key terms for searches, inclusion/exclusion criteria, restrictions by date, can you supply a full list of the international organizations you searched? The current description doesn’t provide enough detail to make clear how the research was conducted.
Author’s response: Please see the answer 2 points below that responds to this comment (starting with “I have

expanded the Context Analysis section…”).

• Similarly, there is insufficient description of the literature review process for it to be replicable. I suggest consulting with literature review reporting guidance (e.g. PRISMA), to check you’ve included all that might be expected for this type of work.
Author’s response: Please see the answer 1 point below that responds to this comment (starting with “I have expanded the Context Analysis section…”).

• If a light touch approach was taken to either the policy review and/or the literature review that would make addressing the two points above difficult, you should consider reporting this differently e.g. a rapid review of policy/literature to inform qualitative interviews. But I think addressing the above two points would be preferable.
Author’s response: We have expanded the Context Analysis section to address your suggestions (line 155-218). Specifically on: (1) line 156-157: clearly define what was considered an SSB-related policy (e.g., taxation, labelling, marketing restrictions, health education); (2) line 158-202: provide a full list of international organizations, databases, and government sources consulted, (3) line 207-209: describe the search terms used (e.g., “sugar-sweetened beverages,” “nutrition,” “child nutrition,” “beverage policy,” “taxation,” “labelling”); (4) line 210: state the time frame (2005–2022) and languages (English and Chinese) for inclusion; (5) line 209-212: clarify inclusion of original research, program reports, and grey literature (identified through expert consultation). In line with your suggestion, I have also specified that our approach was a rapid scoping review rather than a systematic review, given the exploratory purpose of this study and the focus on informing qualitative interviews. I believe this terminology better reflects the methods we used, while ensuring transparency.

• Line 134: “Policy metrics were used to determine the policy environment in China”. Not clear what this means, what policy metrics are you referring to?
Author’s response: We would like to specify that this term refers to policy mapping, where we extracted information and then organized relevant findings. This term has been changed to policy mapping to avoid confusion (line 213-214).

• Line 135: What did the expert consultation consist of?
Author’s response: We have expanded the description of expert consultation, which consisted of suggestions of related grey literature and potential stakeholders for qualitative interviews (line 214-218).

• Public involvement: this sounds more like members of the public were recruited as fieldworkers to distribute recruitment information, rather than as public involvement. Can you explain more the impact of consumers (of SSBs?) holding this roles and/or what impact it had on your research? Also, I’m not sure this is the best location for this section, since you haven’t introduced recruitment of interviewees yet.
Author’s response: This is a requirement by the journal to include a public involvement section. We agree that this might not be the best place to state, as we haven’t introduced the interviewee recruitment yet. This section has been moved to a later section of methods (line 360-363).

• Line 144: Maybe first say who you did interviews with, and who you did focus groups with.
Author’s response: We have made a change to mention the interviewee first, then the interview guide (line 227-235).

• Sentence starting line 146 would be more appropriate for the results section. Appendix A: I’m not sure if “role” is the best descriptor for the third column. Maybe “family role” or “primary caregiver role” or something else?
Author’s response: We left the stakeholder categories (policymakers, nutrition experts, etc.) in this section and moved the number of stakeholders recruited to the results section (line 472-473). For Appendix A, I also changed the descriptor “role” to “primary caregiver role” (Supplementary page 1).

• Line 151: more description of procedure is required e.g. approached by email/phone/something else? What were your eligibility criteria for each group? What information was provided to each type of participant in advance of data collection e.g. participant information sheet? It’s not clear when you switch from talking about consumers to primary care givers whether these are the same or a different group?

Author’s response: We have added a more detailed description of the procedure to approach stakeholders, the eligibility criteria. An information sheet was presented to each participant to introduce the study aim, interview duration, confidentiality assurance, and ethical considerations before the data collection process. Consumers interviewed in this study are primary caregivers of children and adolescents. I have also added more description to make this point clear (line 237-318).

• Line 164: I’m not clear of what a focus group discussion interview is (sounds like two different methods)? Were the focus groups only for consumer groups? How long did focus groups last? If focus groups were indeed used, you should refer to them more clearly throughout, as at the moment I only see mentions of interview hereafter.
Author’s response: Thank you for pointing out that the terminology was unclear. We did not conduct individual interviews with consumers, but rather focus group discussions (FGDs). We have revised the manuscript to consistently use the term FGD instead of “FGD interviews.” We also clarified that FGDs were conducted only with consumer participants, that two FGDs were held in each city (Shanghai and Chongqing), stratified by the age of children (6–11 and 12–17 years), and that each discussion lasted approximately 60 minutes. These clarifications have now been added to the Methods section to avoid confusion (line 275-283, line 316-317).

• Line 177: why was one interview not recorded?
Author’s response: One policymaker didn’t allow recording, and this piece of information has been added (line 317-318).

• Line 180: how was the transcription achieved? By hand/AI/something else?
Author’s response: Transcription was conducted with the assistance of an automatic audio-text converter called Lark Minutes. This detail has been clarified at the beginning of “Data Analysis” (line 325-326).

• Line 193: I’m not sure this needs a full section, could be condensed and subsumed into your analysis section.
Author’s response: This section has been combined with the “Data Analysis” section (line 344-358).

• Line 181: can you explain a bit more about the stages of thematic content analysis? This will help the reader understand what structural codes are etc. Also, I think there’s a typo (familiarisation with?).
Author’s response: I have revised the Data Analysis section to provide a clearer description of the stages of thematic content analysis, as well as correcting the typo. Specifically, we now explain that one analyst (YS) first familiarized herself with the transcripts through repeated reading, and that the coding scheme was initially developed in discussion with QL, the team member with the most qualitative research experience. We further clarify how structural codes were generated deductively from the interview guides, while emergent themes were identified inductively and refined through memo writing and re-coding (line 325-334).

• Sentence starting line 188: think this could go at the start of the methods section.
Author’s response: This sentence is at the start of the Methods section now (line 120-121).

• Line 199: on what date was approval granted?
Author’s response: May 20, 2022. The data has been added to the Ethics Clearance part with in the Methods section (line 366-367).

• It’s not currently clear in your methods how stakeholder mapping was achieved – a description of methodological approach should be added.
Author’s response: We added a description of how stakeholder mapping was achieved, noting that stakeholders were identified based on the context analysis and then mapped across four key sectors in line with consulting an expert in public health nutrition and child nutrition (line 214-218).

• Note that I the interview guides imply the interviews were unstructured to semi-structured – if that’s correct, it would be helpful to add that description to the
Author’s response: We specified that the interviews and focus group discussions were conducted using semi-structured interview guides (line 231-235).

Results

• Signposting at the start of this section could help the reader navigate it e.g. it's divided into the parts to report…etc.

Author's response: Thank you for the suggestion. We have added several lines at the beginning of the Results section to indicate the subsections (line 370-375).

• Section 1.1: while relevant to your study, it's not obvious to the reader how this would be the product of your context analysis, since at the moment the description of methods focuses more on the policy actions (I think the signposting might help).

Author's response: To clarify, I added "As part of the context analysis" at the beginning of section 1.1 (line 388 – 389).

• Line 210: not clear what a P95 value is.

Author's response: This means the 95th percentile value, and a more detailed explanation has been added to the manuscript (line 395).

• Section 1.2: I think appendix d is a clearer articulation of the types of potential policy options. I wonder if this section could be presented in a more visually appealing way e.g. timeline of key policies, nationally and per location? Or a table?

Author's response: We have added a table (Table 1) to list out current SSB-related policies for children and adolescents in China by the title of the policy, issue department, year and the content (page 40).

• Section 2: I don't think the stakeholder mapping is currently very clear. Did you physically map and if so on what software? I think better description in the methods would help.

Author's response: We have added a sentence in the methods section to describe that stakeholder mapping was conducted by listing out after the consultation with the public health expert (line 215-218).

• Section 3: I think line 262 belongs in section 3.

Author's response: Thank you for pointing that out. We have moved the sentence starting from original line 262 to the beginning of section 3 (now line 472).

• Line 269: how was interest/power/position assessed? Not currently clear.

Author's response: Please see Methods – Qualitative Interviews – Data Analysis for an added section on the assessment of interest, power and position, which is part of the stakeholder analysis (line 336 – 342).

• Lines 271 – 276 would be better suited to the methods.

Author's response: This section has been moved to Methods – Qualitative Interviews – Data Collection (starting line 243).

• Section 3: How is this section organised? By theme or something else? Some signposting would help.

Author's response: This section is organized by theme, and we also added several sentences of description at the beginning of section 3 so readers can follow better (line 464-468).

• Line 294 typo – the health the health

Author's response: Corrected (line 506).

• Line 304 typo? Except instead of expect?

Author's response: Yes, it should be except instead of expect. Corrected (line 517).

• Section 3.1.2. – as previously mentioned, the fact that you discuss support for SSB policies means it would be helpful to say up front how those policies were articulated to interviewees.

Author's response: We have updated the Methods – Qualitative Interview – Data Collection section to include how SSB policies were introduced to the participants. Prior to the interview, participants didn't receive any background information as our study would like to capture their unprompted awareness, knowledge and perception towards SSB policies for children and adolescents in China. If the participants showed that they have no prior knowledge on such policies, the interviewer will then explain and introduce

SSB policies to them (line 307-314).

• Line 362 – that the interviews included rankings should be described in the methods, so that here you can simply present the results of the rankings. Also, it would be helpful to know what is meant by adaptability, as this wasn't obvious to me.
Author's response: Corresponding edition was made to add description in the Methods – Qualitative Interview – Data Collection section to indicate that ranking was done during the interview. I also added the definition of "adaptability" here, which combines the consideration of feasibility and effectiveness (line 243-266).

• Figure 2 – how were these scored? How were 0.5 points achieved? How many people responded to these questions?
Author's response: Scores were voted by policy makers and nutrition experts interviewed in this study, they were asked to give a score from 1 to 5, with 1 representing the least adaptable and 5 representing the most adaptable. 0.5 points are shown here because the figure presents an average of scores voted by the participants, and the default scale includes 0.5s. I have made a new figure to make it clearer, and the 0.5s have been removed from the horizontal scale. A total of 11 people responded (line 578).

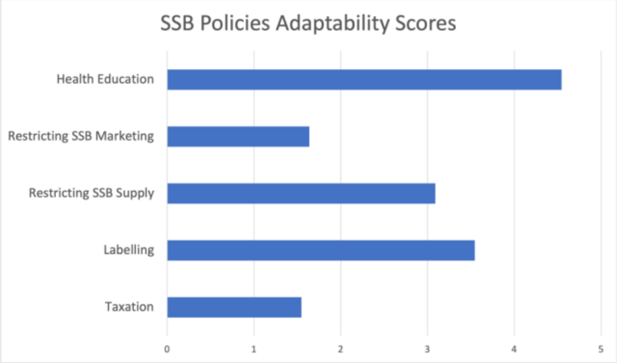

Discussion
• You've made some good reflections on your work, but the discussion could do with some improvement in places. It's currently missing a few things I might expect to see in a discussion, you might want to considering structuring as follows: summary of key findings, strengths and limitations, comparison to existing literature, interpretation, policy implications. In particular, comparing your findings to other comparable research (e.g., qualitative research, not just literature identified in your review), might help interpret your findings in China's context specifically.
Author's response: We agree that highlighting comparisons with comparable qualitative research strengthens the discussion and interpretation. In fact, the original manuscript already included references to qualitative evidence. For example, we compared our findings with Dutch qualitative research showing industry concerns about economic consequences (line 712-714), with international evidence on the role of political will in policy adoption (line 681-683), and with South African qualitative studies documenting industry lobbying tactics and delay strategies (line 718-727). In the revised version, we made the structure of the discussion clearer (summary of findings, strengths and limitations, comparison to literature, interpretation, and policy implications).

• Sentence starting on line 459: I'm not sure that it's obvious why your second clause follows your first?
Author's response: Regarding this sentence, I would like to express that as the government has already issued advocacy policies that demonstrate strong political will, industries might be cautious to be openly opposing governmental actions. According to the industries' reactions during the interview, they are not willing to demonstrate a strong against attitude, but not supportive either, as it is affecting their revenue, they appear to be more natural. I have edited this sentence in a more neutral tone to indicate this scenario observed (line 686).

• The results present a lot of arguments often used by industry to slow the implementation of policies that might affect them, and I agree with your identification/reflection on this in the discussion. I think this could be expanded on further e.g. some might consider introducing step-by-step policies is also an industry tactic!
Author's response: We agree that step-by-step approaches can have dual implications. In the revised manuscript, we have added a sentence to clarify that while incremental or voluntary policy approaches may appear practical, international evidence shows they can also function as industry strategies to delay or dilute regulatory action. We now cite South Africa's documented use of procedural and preparatory delay tactics (line 717-719).

• Paragraph starting 496: you make some good points in this paragraph, but at the moment it's quite hard to follow as it spans a lot of different issues – consider reorganising and/or breaking into smaller paragraphs to aid the reader?
Author's response: Thank you for pointing this out. We have broken this long paragraph into multiple short paragraphs to make it clear to the readers (now line 739 – 770).

• Policy implications section: whilst I don't necessarily disagree with these suggested actions, it's not clear how these are all direct policy implications from your research. Suggest focusing only on those that are directly related to your research. I think this section could also be split into research at and policy implications, which at the moment are quite blended.
Author's response: I have divided the original "Policy Implication" section into "Policy Implication" and "Research Implication", in which policy implication focuses more on the results induce policy suggestions, and research implications elaborate on questions that future research may answer (line 772, 791).

• Limitations: try to draw out more what the limitations of the study might mean for your research i.e. how does evidence mostly collected from urban regions affect the transferability of your findings? Were consumption habits different in COVID and thus you'd find something different if you repeated the study now? Etc
Author's response: I have revised the Limitations section to list how study constraints may influence the study results. Specifically, (1) the predominance of urban participants may limit the transferability of findings to rural populations, where nutrition literacy, consumption habits may be different; (2) COVID-19 may have influenced consumption habits, meaning findings could differ if repeated in a post-pandemic context (line 809-822).

• It would be sensible to reflect on how Coca-Cola's policy influence might also be restricting policy action on SSBs in China. See Susan Greenhalgh's work (here and/or in the discussion) https://www.bmj.com/content/364/bmj.k5050
Author's response: Thank you so much for suggesting this relevant source. I have added this reference at the discussion section where I discussed industry resistance (line 729-720, reference number 57).

*** ***

COI statements:
Reviewer: 1
If you have selected 'Yes' above, please provide details of any competing interests.: Not applicable.

Reviewer: 2
If you have selected 'Yes' above, please provide details of any competing interests.: Not applicable.

VERSION 2 - REVIEW

**Reviewer**	**1**
**Name**	**Niknam, Mahdieh**

Affiliation **Shaheed Beheshti University of Medical Sciences, Community Nutrition**

Date **24-Sep-2025**

COI

Thank you for submitting the revised version of your manuscript. I appreciate the efforts you have made in addressing the comments from the previous review round.

Overall, the manuscript is well prepared, and the conclusions are supported by the data presented. I recommend acceptance of this article.

Reviewer **2**

Name **Forde, Hannah**

Affiliation **University of Oxford, Nuffield Department of Primary Care Health Sciences**

Date **08-Oct-2025**

COI

Well done to the authors for responding so thoroughly to the reviewer comments. I just have a few outstanding comments:

Abstract:

The design section is hard to follow e.g. "from the review and expert consultation" - expert consultation hadn't been introduced before this point. I think this may be an issue of simply switching terminology to be consistent throughout (is expert consultation the same as stakeholder analysis, which you have mentioned?). Also, it could be clearer to refer to the precise method you collected your data (focus groups and interviews?) at some point in this section.

Line 23: referring to thematic analysis would be more precise.

Line 26 onwards: not clear if the sentence starting "A comprehensive national standard.." was reported in your focus groups or is something you've concluded as a result of the entirety of your research (at the moment it reads a bit more like a conclusion).

Interested that you've concluded that raising health knowledge is what's needed to advance SSB policy introduction, given that changing standards would probably have a greater effect (if that's what your data concluded then no need to change it, just intrigued!).

Article summary:

You refer to only interviews, even though I believe you also conducted focus groups?

Introduction:

Line 101: thanks for clarifying which policies you're referring to. Based on the policies you've listed, I think you're interested in policies that discourage rather than restrict SSB consumption, so you could amend the sentence accordingly. I think you can delete the sentence starting "Unrestricted SSB intake…" as you've already made that case earlier in the introduction.

I think you could articulate more clearly exactly why understanding stakeholder perspectives is important – I don't think it's enough to say that it's not been done before. What would stakeholder views offer? How would a greater understanding those views affect future research/policy? Consider taking a look at other papers that have conducted comparable research in different countries/contexts to see how they justified their study.

Methods:

Well done for successfully applying changes to this section, it reads much better.

Note that the name of the framework you refer to in this section is different to the one you refer to in the abstract.

Line 144: it's not clear if your definition only includes tax/labelling/marketing/health education campaigns, or if these are examples of the kinds of policies you were interested in – I think a minor wording change would fix this.

Line 145: I think this sentence is missing something – policies were collated/searched for in these sources, something like that? And roughly when did you search these sources? That will help the reader understand what policies were implemented at that time.

Line 162: when you say expert consultation, do you mean the experts you later consult with formally in your study, or someone else? Either way fine, just would be clearer to clarify.

Line 163: What are policy metrics? Please define

Line 165: Should be the start of a new paragraph? I think these sentences would fit better under the qualitative interview sub-header.

Thank you for adding greater detail to the qualitative interview section, it us a much more thorough account of your research.

Line 199: this is the clearest definition so far of your policies of interest, I think situating this earlier in the paper (e.g. when you first refer to your policies of interest), would mean you needn't repeat them so often throughout the text.

Line 259: were the translations conducted by the authors/software, and did you verify the translations in any way?

Line 279: I think you may be referring to snowball sampling, could say as such

Results

The signposting at the beginning of this section is really helpful.

At the end of the methods section you refer to conducting stakeholder analysis, but I can't see any mentions of the findings of that in the results section (except as a header for the thematic analysis)? I'm not clear if or how the two analysis methods have been blended together – I think perhaps you used the stakeholder analyses within your thematic analysis to compare across stakeholder groups (line 374; if so, sounds like Gale & co's Framework Method), but I'm not sure?

Line 379: think this sentence would sit batter at the very beginning of section 3

Discussion

Overall, your discussion is a lot stronger than it was previously, well done.

Line 562: I'm not sure what this sentence means/adds, and I wouldn't say it was a key finding of your study – indeed your introduction already makes the case that SSB policies are needed for health reasons.

In the paragraph starting line 643 (or somewhere else in the discussion if you think it would fit better), I think it could be helpful to interpret/compare to existing literature your finding that some stakeholders didn't think SSB taxes or marketing restrictions wouldn't be effective.

Line 706: Would be better to make clear earlier in the study that you're only interested in packaged SSBs before you reflect on it here.

VERSION 2 - AUTHOR RESPONSE

Reviewer: 1
Ms. Mahdieh Niknam, Shaheed Beheshti University of Medical Sciences
Comments to the Author:
Thank you for submitting the revised version of your manuscript. I appreciate the efforts you have made in addressing the comments from the previous review round.

Overall, the manuscript is well prepared, and the conclusions are supported by the data presented. I recommend acceptance of this article.

Response: Thank you so much for your comment! We truly appreciate it.

Reviewer: 2
Dr. Hannah Forde, University of Oxford
Comments to the Author:
Well done to the authors for responding so thoroughly to the reviewer comments. I just have a few outstanding comments:

Abstract:
The design section is hard to follow e.g. "from the review and expert consultation" - expert consultation hadn't been introduced before this point. I think this may be an issue of simply switching terminology to be consistent throughout (is expert consultation the same as stakeholder analysis, which you have mentioned?). Also, it could be clearer to refer to the precise method you collected your data (focus groups and interviews?) at some point in this section.

Response: Thank you for the comment. Before we moved to qualitative interviews, we identified who are the stakeholders engaging in this public health question and who we would

like to interview, and this step was defined as “stakeholder mapping”. The identification of interviewed stakeholders come from context analysis, which has two parts: (1) policy and literature reviews, (2) we consulted a public health nutrition expert to validate and refine the list of stakeholders. Therefore, we used this phrasing to reflect these steps (line 8-12). We also made amendments in the main text “Methods” section to clarify this procedure involving expert consultation (line 187-189, line 196-197).

Besides, “qualitative interviews” was used as an umbrella term that includes multiple forms, such as in-depth interviews and focus group discussion.

Line 23: referring to thematic analysis would be more precise.

Response: We appreciate your suggestion to refer explicitly to the thematic analysis. However, the sentence was intended to describe the empirical insights from the qualitative interviews rather than to emphasize the analytical method. Therefore, we revised line 23 from “Qualitative results” to “Qualitative interviews” (line 24).

Line 26 onwards: not clear if the sentence starting "A comprehensive national standard.." was reported in your focus groups or is something you've concluded as a result of the entirety of your research (at the moment it reads a bit more like a conclusion).

Response: Mutiple stakeholders has mentioned this point (including policy makers, nutrition experts and industry). Instead, we edited line 40 to start with “multiple stakeholders mentioned that…” to make it clear that this is a result of the qualitative interviews.

Interested that you've concluded that raising health knowledge is what's needed to advance SSB policy introduction, given that changing standards would probably have a greater effect (if that's what your data concluded then no need to change it, just intrigued!).

Response: Given the current results, we think both actions are needed, while raising health knowledge might be a precursor step ahead of changing the national standard as we believe that awareness has to be reached first then inducing a policy/standard change. Stakeholders have mentioned this point because in the Chinese context, a national standard would facilitate any type of policy roll-out more easily.

Article summary:
You refer to only interviews, even though I believe you also conducted focus groups?

Response: Thank you for mentioning this. In this study, we used three forms of qualitative interviews, including key informant interviews, in-depth interviews, and focus group discussion to gather qualitative findings. We have made some edits in line 55 to clarify this.

Introduction:
Line 101: thanks for clarifying which policies you’re referring to. Based on the policies you’ve listed, I think you’re interested in policies that discourage rather than restrict SSB

consumption, so you could amend the sentence accordingly. I think you can delete the sentence starting "Unrestricted SSB intake…" as you've already made that case earlier in the introduction.

Response: We have changed that sentence to "However, China has not announced a national policy directly discouraging SSB consumption" (line 113-114). Besides, the sentence starting "unrestricted SSB intake…" has been removed (line 121).

I think you could articulate more clearly exactly why understanding stakeholder perspectives is important – I don't think it's enough to say that it's not been done before. What would stakeholder views offer? How would a greater understanding those views affect future research/policy? Consider taking a look at other papers that have conducted comparable research in different countries/contexts to see how they justified their study.

Response: We have added more lines to justify why understanding stakeholder perspectives is necessary rather than stating nobody has done this before (line 115-121). Stakeholder perspectives would essential information to collect when establishing a brand new policy as this enables us to understand the policy environment better, thus facilitating policy formulation and implementation.

Methods:
Well done for successfully applying changes to this section, it reads much better.

Response: Thank you so much for your advice to make this section sounds clear!

Note that the name of the framework you refer to in this section is different to the one you refer to in the abstract.

Response: Thank you for pointing this out. The names of the framework have been edited to a consistent form of expression (edits in line 7 and 53).

Line 144: it's not clear if your definition only includes tax/labelling/marketing/health education campaigns, or if these are examples of the kinds of policies you were interested in – I think a minor wording change would fix this.

Response: We clarified this line by stating taxation, labelling, marketing restrictions and health education campaigns were examples of the types of SSB policies that we focused and included in our review (line 163-164).

Line 145: I think this sentence is missing something – policies were collated/searched for in these sources, something like that? And roughly when did you search these sources? That will help the reader understand what policies were implemented at that time.

Response: We added some details saying policies were collected from these sources (starting line 164), and specified the period during which the search was conducted (line 180-181).

Line 162: when you say expert consultation, do you mean the experts you later consult with formally in your study, or someone else? Either way fine, just would be clearer to clarify.

Response: The expert we consulted for stakeholder mapping was also interviewed later on in the later qualitative interviews. At the time of consultation, the expert provided preliminary input to refine the stakeholder we mapped and identify potential interviewees. Their participation in the qualitative interview was part of the formal data collection process, following informed consent. We also clarified this point in the manuscript (line 187-188).

Line 163: What are policy metrics? Please define

Response: We have revised to add more description on policy metrics (line 191-194). Specifically, we stated the data extraction process we did to summarize key features of each policy, including the title, issuing department, year of issue, and policy content. These extracted metrics were then integrated to compare current SSB-related policies for children and adolescents in China.

Line 165: Should be the start of a new paragraph? I think these sentences would fit better under the qualitative interview sub-header.

Response: We have made the section starting “Based on the context analysis results” (line 196).

Thank you for adding greater detail to the qualitative interview section, it us a much more thorough account of your research.

Response: Thank you so much for your comment.

Line 199: this is the clearest definition so far of your policies of interest, I think situating this earlier in the paper (e.g. when you first refer to your policies of interest), would mean you needn’t repeat them so often throughout the text.

Response: Thank you for the suggestion. We have moved this specific definition to the context analysis part, where we first mentioned these five forms of SSB policies we focused in this study (line 159-164). The repeated explanation in this part has been deleted (line 226-227).

Line 259: were the translations conducted by the authors/software, and did you verify the translations in any way?

Response: We have added more explanation on how the translation was conducted and verified (line 278-279).

Line 279: I think you may be referring to snowball sampling, could say as such

Response: We guessed you meant the section of “Patient and public involvement”? It is a section required by the journal to state the engagement of public during the design of the study.

Results
The signposting at the beginning of this section is really helpful.

Response: Thank you so much for your comment.

At the end of the methods section you refer to conducting stakeholder analysis, but I can’t see any mentions of the findings of that in the results section (except as a header for the thematic analysis)? I’m not clear if or how the two analysis methods have been blended together – I think perhaps you used the stakeholder analyses within your thematic analysis to compare across stakeholder groups (line 374; if so, sounds like Gale & co’s Framework Method), but I’m not sure?

Response: We clarified the role of stakeholder analysis in the study. In the Methods section, we added some sentences explaining what stakeholder analysis is, and describing how it identifies actors, interests, and power relations (line 282-285). In the Results section, we revised the introduction sentence and section “3. Stakeholder Analysis from Qualitative Interviews” to show that stakeholder analysis integrated findings from context analysis, stakeholder mapping, and qualitative interviews (line 387-388). Thematic analysis is an analytical method we used to interpret qualitative data.

Line 379: think this sentence would sit batter at the very beginning of section 3

Response: We guess you are referring to the sentence “In this study, we included 9 policymakers, 2 nutrition experts, 5 industry stakeholders and 11 consumers…” at the beginning of section 3.1? We have moved it to the beginning of section 3 now (line 394-401).

Discussion
Overall, your discussion is a lot stronger than it was previously, well done.

Response: Thank you so much.

Line 562: I’m not sure what this sentence means/adds, and I wouldn’t say it was a key finding of your study – indeed your introduction already makes the case that SSB policies are needed for health reasons.

Response: This sentence has been deleted (line 584).

In the paragraph starting line 643 (or somewhere else in the discussion if you think it would fit better), I think it could be helpful to interpret/compare to existing literature your finding that some stakeholders didn’t think SSB taxes or marketing restrictions wouldn’t be effective.

Response: We revised the Discussion to compare our findings with existing literature (line 666-681). Evidence from the Netherlands and Iran was added to show that similar doubts about the effectiveness of SSB taxes and marketing restrictions were also observed among stakeholders elsewhere. We further explained that differences in study populations may account for these inconsistencies, as modelling studies show higher price responsiveness among low-income groups. In addition, we added new empirical evidence on substitution effects in China, showing increased caloric intake despite reduced SSB purchases (line 686-691).

Line 706: Would be better to make clear earlier in the study that you're only interested in packaged SSBs before you reflect on it here.

Response: We made a correction stating in this study we focus on policies that discourage the intake of packaged SSBs (line 160).

VERSION 3 - REVIEW

**Reviewer**	**2**
**Name**	**Forde, Hannah**
**Affiliation**	**University of Oxford, Nuffield Department of Primary Care Health Sciences**
**Date**	**11-Nov-2025**
**COI**	

Again, the authors have done an excellent job of responding to comments, I especially liked the lines you added to the introduction to justify conducting a study to understand stakeholder perspectives. My only remaining suggestion to make is that I don't think qualitative interviews is an accurate umbrella term that would encompass focus group discussions; focus groups are a distinct methodology, so I suggest rewording accordingly.

VERSION 3 - AUTHOR RESPONSE

Reviewer: 2
Dr. Hannah Forde, University of Oxford
Comments to the Author:
Again, the authors have done an excellent job of responding to comments, I especially liked the lines you added to the introduction to justify conducting a study to understand stakeholder perspectives. My only remaining suggestion to make is that I don't think qualitative interviews is an accurate umbrella term that would encompass focus group discussions; focus groups are a distinct methodology, so I suggest rewording accordingly.

Author's response: Thank you so much for your comments.

Regarding the description of qualitative interviews, we made changes in the abstract and the article summary to make it sound better.

1) Asbtract (line 11-12): We specified that we mapped. stakeholders for key informant interview, in-depth interview, and focus group discussions to explore stakeholders' perceptions of SSB policies
2) Article summary (line 42-45): We stated that we applied qualitative data collection (which including key informant interview, in-depth interview, focus group discussions) for this study.
